# Targeted Multiresidue Method for the Analysis of Different Classes of Pesticides in Agro-Food Industrial Sludge by Liquid Chromatography Tandem Mass Spectrometry

**DOI:** 10.3390/molecules26226888

**Published:** 2021-11-15

**Authors:** Niki C. Maragou, George Balayiannis, Evangelos Karanasios, Emilia Markellou, Konstantinos Liapis

**Affiliations:** 1 Laboratory of Chemical Control of Pesticides, Scientific Directorate of Pesticides’ Control and Phytopharmacy, Benaki Phytopathological Institute, 14561 Athens, Greece; g.balayiannis@bpi.gr; 2 Laboratory of Environmental Control of Pesticides, Scientific Directorate of Pesticides’ Control & Phytopharmacy, Benaki Phytopathological Institute, 14561 Athens, Greece; e.karanasios@bpi.gr; 3 Laboratory of Mycology, Scientific Directorate of Phytopathology, Benaki Phytopathological Institute, 14561 Athens, Greece; e.markellou@bpi.gr; 4 Laboratory of Pesticide Residues, Scientific Directorate of Pesticides’ Control and Phytopharmacy, Benaki Phytopathological Institute, 14561 Athens, Greece; k.liapis@bpi.gr

**Keywords:** fungicides, insecticides, agricultural products, method optimization, extraction, environmentally friendly, sustainability, industrial waste

## Abstract

Sludge generated after washing of fruits and vegetables during agro-food processes is a complex matrix and selective methods for the identification and quantification of pesticides’ residues are necessary in order to achieve a sustainable and effective management of the total sewage. The present work describes the development and validation of a reliable, simple and fast analytical method based on liquid chromatography-tandem mass spectrometry (LC-MS/MS) for the determination of 47 pesticides of different chemical classes, including organosphosphates, pyrethroids, neonicotinoids, triazoles and others, in sludge samples after QuEChERS sample preparation. The necessity of the individual steps of QuEChERS was investigated and the LC-ESI-MS/MS conditions were optimized to achieve maximum sensitivity of the target analytes. The method limits of detection (LODs) ranged between 0.0005 mg/kg (imidacloprid) and 0.05 mg/kg (beta cyfluthrin). The recoveries ranged between 71–120% and the repeatability of the method was ≤25% expressed as relative standard deviation. The method was applied to sludge samples generated after washing of fruits in an agro-fruit-packaging unit in Greece. The results showed the presence of 37 pesticides’ active substances with concentrations ranging from low ppbs, such as fludioxinil (5 μg/kg) up to low ppms such as beta cyfluthrin (3.5 mg/kg) and with their sum concentration reaching up to 19 mg/kg.

## 1. Introduction

Agro-food processes usually concern washing of fruits and vegetables before packaging and distribution to the market. The generated effluent, apart from the aqueous phase, includes sludge coming from the soil and other material from the field, such as leaves and pieces of wood that are transferred with the plastic containers. In order to achieve a sustainable and effective management of the total effluent of such a procedure it is important to be aware of the pesticides’ content not only in the aqueous phase but in the sludge as well. However, sludge is a complex and difficult matrix to analyze, and suitable and reliable methods are required for the determination of contaminants present at low levels such as pesticides.

Complex matrices such as sludge usually involve labor-intensive extraction and clean-up procedures before the chromatographic analysis [1]. Soxhlet and pressurized liquid extraction (PLE) have been applied for the determination of organochlorine pesticides in sewage sludges followed by silica/aluminum oxide clean-up and GC-ion trap MS/MS analysis [2]. Matrix solid-phase dispersion (MSPD) has been applied for the determination of fifteen pesticides belonging to the organophosphate, triazine, organochlorine, triazole, pyrethroid, carbamate and other chemical groups in drinking water treatment sludge followed by GC-MS analysis [3]. The QuEChERS extraction (quick, easy, cheap, effective, rugged and safe) has recently been applied to limited studies regarding the determination of pesticides in sludge samples. In particular QuEChERS was applied to sewage sludge generated from agro-food industry [4], in sludge obtained from wastewater treatment plant [5] and in sludge from a pilot plant [6] in all cases followed by LC-MS/MS. Comparison between PLE and QuEChERS showed that the second was superior for samples of sediment, soil and sludge [5].

Since it was first introduced, the advantages of the QuEChERS procedure have been a strong motive for analytical scientists to transfer this “revolutionary” preparation method from fruits and vegetables to as many matrices as possible [7,8]. However, in the case of matrices where analyte-matrix interactions are stronger, the efficiency of the method had to be thoroughly investigated. In soil, stronger extraction conditions have historically been applied (pressurized liquid extraction (PLE) or soxhlet extraction, etc.), rather than just shaking, in order to overcome the strong binding characteristics of the matrix [8]. A number of applications of the QuEChERS method in soil has been published for the determination of a wide range of pesticides demonstrating the advantages of QuEChERS in terms of low cost, simplicity, short extraction time and low organic solvent consumption, offering at the same time satisfactory analytical features [9,10,11,12,13,14,15,16]. Apart from sludge [4,5,6] and soil [9,10,11,12,13,14,15,16] QuEChERS has been applied to other particular matrices such as cotton-based textile [17]. However, these applications mainly involve compounds prone to gas chromatography [9,10,11,13,16,17], while the liquid chromatographic determinations include up to 25 analytes [15]. Furthermore, it is noted that since the QuEChERS extraction includes multiple steps that differentiate according to the physicochemical properties of the analytes and the matrix, all these applications include numerous modifications made by almost each user, depending on the scope of each method.

The aim of the present work was the development of a QuEChERS based sample preparation followed by LC-ESI-triple quadrupole MS/MS method after a stepwise optimization, which allows the simultaneous determination of a large number of pesticides that belong to different chemical classes in sludge at the low ng/g level. The present work improves the coupling of QuEChERS–LC-MS/MS including pesticides with different physicochemical properties, with LogPow values between −0.24 to 7.02 and including for the first time the group of pyrethroids in the liquid chromatographic analysis of the complex matrix of sludge.

## 2. Materials and Methods

### 2.1. Chemical and Reagents

The target compounds mainly concern fungicides and insecticides, and they belong to the chemical classes of organosphosphates, pyrethroids, neonicotinoids, triazoles, benzimidazoles, pyridine compounds, pyrimidines, strobilurins, anilides, pyrazoles, diacylhydrazines, carbamates, diphenyl oxazolines, oxadiazines, pyrroles, sulfite esters and tetramic acids.

High purity (96.2–99.9%) individual standards of the 47 selected pesticides and the internal standard triphenyl phosphate (99.5%), were purchased from Chem Service (West Chester, PA, USA), Dr Ehrenstorfer (Augsburg, Germany), FMC (Shangai, China) and Sigma Aldrich, (Seelze, Germany). The target compounds, their molecular weight, formula, structure, function and partition coefficient (pKow) are listed in Table S1 (Supplementary Materials) [18,19].

HPLC water and HPLC methanol (MeOH) were purchased from Fischer Scientific (Leicestershire, UK) and HPLC acetonitrile (ACN) was purchased from CARLO ERBA (Val de Reuil, France). Dimethyl formamide (DMF) was obtained from Panreac Quimica (Barcelona, Spain). Individual stock standard solutions 1000 μg/mL of the internal standard and of each analyte were prepared in acetonitrile, except for chlorantraniliprole which was dissolved in methanol and carbendazim which was dissolved in ACN:DMF (1:1, *v/v*). The stock solutions were used for further dilutions (mix standard solutions of 100, 10 and 1 μg/mL) for linearity and recovery experiments. Syringe driven Polyester (PET) filters with pore size 0.20 μm and 15 mm diameter (Macherey-Nagel, Allentown, PA, USA) were used for the filtration of aqueous based standard and sample solutions and syringe driven Nylon filters with pore size 0.22 μm and 25 mm diameter (Macherey-Nagel, Allentown, PA, USA) were used for the filtration of totally organic solvent standard and sample solutions before injection.

For the sample preparation 50 mL Falcon tubes containing 4 g magnesium sulfate (MgSO_4_), 1 g sodium chloride (NaCl), 1 g sodium citrate tribasic dehydrate (Na citrate) and 0.5 g sodium citrate dibasic sesquihydrate (Na citrate sesquihydrate) and 15 mL Falcon tubes containing 900 mg magnesium sulfate (MgSO_4_) and 150 mg primary secondary amine (PSA) were purchased from Interchim (Montluçon, France). A Techne sample concentrator (Staffordshire, UK) was used for the evaporation of solvent using a gentle nitrogen stream.

### 2.2. Sample Collection

Sludge samples were collected from an agro-fruit-packaging unit in Greece in March 2019. A washing tank was used to wash fruits collected from the field. After the completion of the washing process the tank was emptied and the washing effluents were collected in a separate tank where they were stored until further treatment. Four samples from the sludge remained in different points of the washing tank, as well as one sample of the sludge of the collecting tank were sampled. The sampling was conducted with a shovel in unused plastic bags and stored at −20 °C until analysis.

### 2.3. Sample Preparation

**Figure 1:**
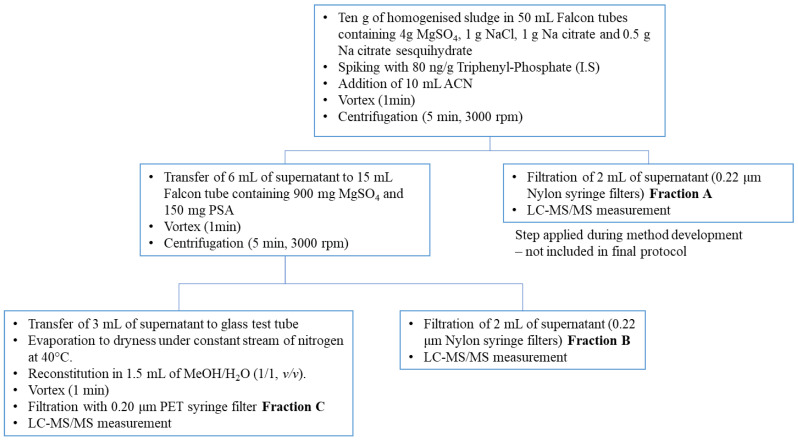
Method protocol.

After the samples were defrosted, they were homogenized manually with a spoon. The sample preparation followed was based on QuEChERS method [20] which concerns extraction with acetonitrile and clean-up by dispersive solid phase extraction in two steps. Figure 1 describes the steps of the sample preparation procedure. Ten grams of homogenized sludge were weighed directly in 50 mL Falcon tubes containing 4 g MgSO_4_, 1 g NaCl, 1 g Na citrate and 0.5 g Na citrate sesquihydrate and spiked with 100 μL of 8 μg/mL of the internal standard triphenyl-phosphate. For the recovery study and the method validation, the samples were additionally spiked with the appropriate aliquot of 1 or 10 μg/mL mix standard solution. Next, 10 mL ACN were added and immediately the Falcon tube was vortexed for 1 min and centrifuged for 5 min at 3000 rpm (revolutions per minute). Two mL of the supernatant were filtered with 0.22 μm Nylon syringe filters and measured by LC-MS/MS. This fraction of the sample was named Fraction A, and was evaluated during method development. Six mL of the same supernatant were transferred to the 15 mL Falcon tube containing 900 mg MgSO_4_ and 150 mg PSA for the second extraction step. The tube was vortexed for 1 min and centrifuged for 5 min at 3000 rpm. Two mL of the supernatant were filtered with 0.22 μm Nylon syringe filters syringe filters and measured by LC-MS/MS (Fraction B). Three mL of the same supernatant were transferred to a glass test tube and evaporated to dryness under a constant stream of nitrogen at 40 °C, finally the extract was reconstituted in 1.5 mL of MeOH/H_2_O (1/1, *v/v*) followed by one-minute vortex stirring (Fraction C) and filtered with 0.20 μm PET syringe filter before LC-MS/MS measurement. In every batch of six sludge samples a procedural blank was also prepared according to the same procedure followed for the samples but weighing 10 g of water instead of sludge sample. All the target analytes were determined and quantified in the three collected Fractions (A, B and C) of spiked sludge samples. Qualitative and quantitative evaluation of the results obtained from the three fractions was performed for the final selection of the method protocol.

The determination of moisture of sludge samples was performed by weighing accurately an amount of sample of approximately 4–5 g and drying in an oven at 100 °C until stable weight.

### 2.4. LC-MS/MS Measurements

#### 2.4.1. LC-MS/MS Optimization

The LC-MS/MS measurements were carried out using a Varian 1200L Prostar LC/MS triple quadrupole with an electrospray ionization interface (ESI) in positive and negative polarity (Walnut Creek, California, USA). Varian MS Workstation software version 6.8 was used for the data recording and analysis including peak integration. Chromatographic separation was performed with a Kinetex C18 analytical column (50 × 2.10 mm) with particle size 2.6 μm and pore diameter 100 Å, equipped with a guard C18 column (2 × 2.10 mm).

The optimization of the LC-MS/MS conditions was based on the strategy plan proposed in previous work [21].

The first step was the selection of the parent mass /masses and their capillary voltage and the selection of the product ions and their collision energies (4 SRMs selected for each compound). In particular, stock standard solutions of each compound diluted in methanol (1–5 μg/mL) were measured by flow injection analysis (FIA) at 50 μL/min flow rate for the selection of the ionization polarity, the parent masses, the optimization of the capillary voltages, the selection of the product ions and the optimization of the collision energies. Full scan mode (MW ± 100 amu) was applied for the selection of the ionization polarity and parent masses, which was based on the highest abundance. After the selection of the ionization polarity and parent mass, the capillary voltage that enhances its ionization was optimized and the breakdown curve was generated. Four selected reaction monitoring transitions (SRM) were selected for each compound and their collision energies were optimized. Based on these results the two transitions with the highest sensitivity were selected for each compound for the LC-MS/MS method as quantification (SRM1) and confirmation (SRM2) transitions.

Next, the analytes were grouped in eight mixed solutions of five to eight compounds, based on their structure and polarity, in order to investigate their chromatographic behavior and complete the optimization of the LC-MS/MS parameters. The mixed solutions were injected separately to the analytical column and four SRMs were recorded for each compound.

The starting point of the gradient elution was based on the European Standard EN 15662 [20], but since the analytes exhibited very different chromatographic behavior further optimization of the gradient was required in order to have good chromatography in terms of sensitivity (signal to noise ratio), resolution and analysis time. It is noted that the scope of the gradient was to spread the eluted chromatographic peaks throughout the analysis time so that all segments of the MS/MS method to include the least possible compounds, so that the data points to be adequate (>10 points) for quantification. Based on these experiments and on the retention time of the analytes, twenty time-segments of one minute were created for the MS/MS method and each SRM was recorded only for three consecutive time segments (three minutes) based on the retention time of the target compound. The appropriate dwell and scan time was selected for each segment based on the number of monitored transitions so that adequate number of data points were recorded for each chromatographic peak.

After the selection of the optimum gradient, it was considered appropriate to optimize the drying gas temperature. In particular, because of the fact that the gradient starts with high content of aqueous mobile phase, 90%, which decreases to low content of 10% until the elution of the last peaks, optimization experiments were conducted for the drying gas temperature examining a wide range of temperatures with highest value 340 °C, lowest value 200 °C, and intermediate value 280 °C, while a ramp program starting at 340 °C, remaining stable for the first three minutes and decreased to 200 °C until the 16th min in steps of 10 °C was also tested.

#### 2.4.2. LC-MS/MS Final Conditions

The optimum gradient elution applied consisted of a mobile phase composed of 5 mM ammonium formate and 0.1% *v/v* formic acid in methanol (solvent A) and 5 mM ammonium formate and 0.1% *v/v* formic acid in water (solvent B) at 0.25 mL/min flow rate. The gradient starts at A/B 90/10, remains for 0.5 min and changes linearly to A/B 10/90 in 11.5 min where it remains stable until the 20th minute. Afterwards the composition of the gradient returns to the initial ratio A/B 90/10 and remains stable for 10 min for equilibration. The column oven was set at 40 °C and the injection volume at 5 μL.

Electrospray (ESI) in positive polarity was applied for the 46 pesticides and ESI in negative polarity for Fludioxonil. Data were acquired in SRM mode with two transitions per analyte and one transition for the internal standard. Table 1 presents the SRMs of the target compounds and the internal standard, ordered by their retention time, along with the capillary voltage (CV) and collision energy (CE) of each transition. The acquiring MS/MS method was divided in twenty (20) time-segments of one minute, except for the last segment (10 min) and the SRMs of each analyte were recorded only for three consecutive time-segments based on the retention time of the analyte. The drying gas temperature was set at 340 °C for the first three minutes and decreased to 200 °C until the 16th min in steps of 10 °C. The number of SRMs included in each time-segment, the dwell time of each transition, the total scan time of each time-segment and the drying gas temperature set for each time-segment are presented in Table S2 (Supplementary Materials). The drying gas pressure (N_2_) was set at 19 psi and the nebulizing gas pressure at 55 psi. The needle voltage was held at 5 kV. All the working LC-MS/MS conditions are the same for the positive and the negative polarity.

### 2.5. Method Validation

The linearity of the response of the LC-ESI-MS/MS system versus analyte concentrations was examined with standard calibration curves prepared in acetonitrile for the quantification of the analytes contained in Fractions A and B and in methanol/water (1/1, *v/v*) for the quantification of the analytes contained in Fraction C. Five standard solutions were measured with concentration levels ranging between 1 and 500 ng/mL for all the analytes. The working solutions in ACN and methanol/water (1/1, *v/v*) contained also 100 and 200 ng/mL of triphenyl phosphate as internal standard, respectively.

Matrix matched calibration curves were prepared with sludge samples spiked with the target analytes at four levels between 1–200 ng/g and with triphenyl phosphate at 100 ng/g. Spiked samples and non-spiked samples of the same sampling point were analyzed according to the protocol described in Section 2.3 “Sample preparation”. The final linear equation of the matrix matched calibration curve resulted after the subtraction of the signal of the unfortified sample from the signal of the fortified samples.

For the assessment of the overall precision and accuracy, the method was applied to sludge sample that was spiked with the target analytes at four fortification levels (1, 10, 50 and 200 ng/g) and analyzed in five replicates. The recovery (%R) of the method was calculated by subtracting the concentration measured in the non-spiked sample from that measured in the spiked sample and then dividing with the spiked concentration (C_ADDED_) according to Equation (1).

(1) $$\%R\;=\;\frac{C_{\mathrm{SPIKED}\;\mathrm{SAMPLE}}-C_{\mathrm{NONSPIKED}\;\mathrm{SAMPLE}}}{C_{\mathrm{ADDED}}}\times 100$$

The method limit of detection (LOD) for the different analytes was defined as the concentration of the analyte in matrix that was equal to three times the average level of the baseline noise close to the peak. The method limit of quantification (LOQ) was defined as the lowest validated concentration with acceptable recovery and repeatability. The acceptability criteria were based on the guidance document SANTE/2020/12830, Rev.1 [22] which reports a general requirement for the mean recovery to be in the range of 70–120% and the precision to be ≤20% expressed as relative standard deviation (RSD), independently the sample matrix and the concentration level. However, it is noted that for difficult matrices such as food of plant and animal origin the acceptability criteria for precision at low concentration levels ≤ 10 ng/g extends to 30% in the same guidance document. Sludge is considered a difficult matrix and the same acceptability criteria could be adopted.

## 3. Results and Discussion

### 3.1. Optimization of LC-ESI-MS/MS

**Figure 2:**
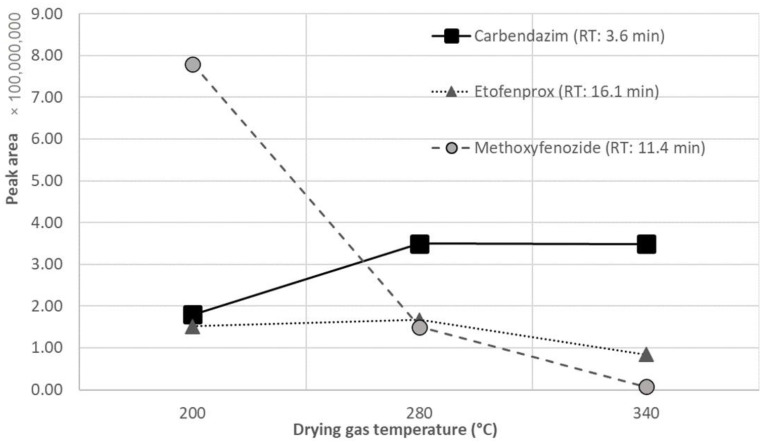
Effect of drying gas temperature in ESI on the sensitivity (as peak area) of three different analytes (carbendazim, methoxyfenozide and etofenprox). RT: retention time ×.

The optimization of the drying gas temperature during electrospray ionization proved to be a critical parameter. Figure 2 illustrates the peak area of the chromatographic peak of three indicative analytes (carbendazim—early eluting, methoxyfenozide—intermediate eluting, etofenprox—late eluting) obtained at three temperatures of drying gas, where different patterns are observed for the different analytes. It is shown that for the early eluting carbendazim with Retention Time (RT) at 3.6 min, the higher the drying gas temperature the higher the signal of the peak area, whereas for methoxyfenozide which elutes in the middle of the chromatographic run (RT: 11.4 min) and etofenprox which is one of the last eluting compounds (RT: 16.1 min) lower temperature for the drying gas is required. The change of the sensitivity in relation to the drying gas temperature follows the gradient of the mobile phase as regards the content of water. The higher the content of water in the mobile phase the higher temperature is required for the solvent to evaporate during the electrospray ionization. Based on these data a gradient program of drying gas was applied in order to achieve optimum sensitivity of all analytes (Table S2 in Supplementary Materials).

### 3.2. Optimization of the Sample Preparation

**Figure 3:**
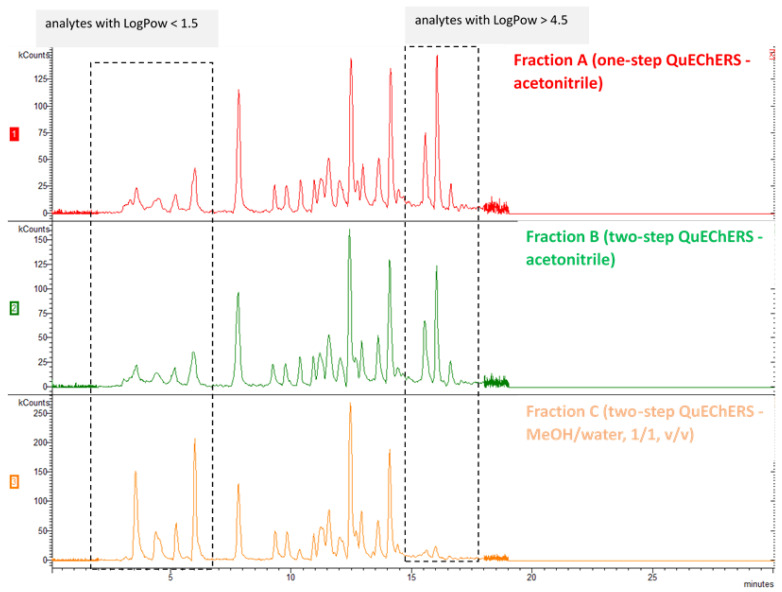
Comparison of TIC chromatograms of Fraction A (red line), Fraction B (green line) and Fraction C (orange line) of a sludge sample fortified with all the analytes at 10 ng/g and internal standard at 100 ng/g.

**Figure 4:**
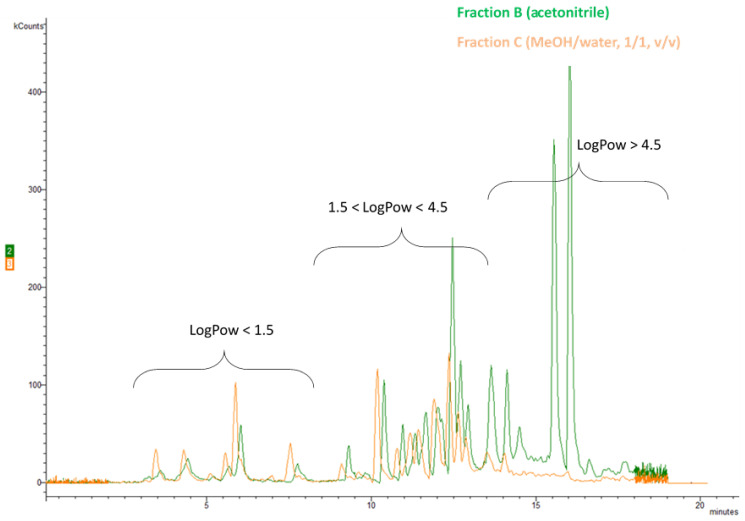
Comparison of TIC chromatograms of Fraction B (green line) and of Fraction C (orange line) of a sludge sample fortified with all the analytes at 10 ng/g and internal standard at 100 ng/g.

The final method protocol is illustrated in Figure 1. Qualitative evaluation of chromatograms obtained from the three different Fractions A, B and C from the sample preparation revealed that in Fractions A and B the signal of the early eluting analytes (with RT < 8 min) was significantly lower than the corresponding signal of Fraction C. At the same time the signal of the late eluting analytes (with RT > 15 min) was significantly lower in Fraction C comparing to the signal obtained from Fractions A and B. This comparison is illustrated in Figure 3 with the Total Ion Chromatograms of Fraction A, B and C of the same fortified sludge sample at 10 ng/g. This can be attributed to the fact that the analytes in Fraction C are dissolved in MeOH/water (1/1, *v/v*) whereas the analytes in Fractions A and B are dissolved in acetonitrile. The results show that for compounds with low partition coefficient (logPow < 1.5), which concern the early eluting analytes flonicamid, carbendazim, thiamethoxam, thiabendazole, clothianidin, imidacloprid, acetamiprid, thiacloprid and thiophanate methyl the solvent the most appropriate is MeOH/water (1/1, *v/v*). On the other hand, for analytes with high partition coefficient (logPow > 4.5) the organic solvent acetonitrile significantly improves the chromatography and potentially the dissolvation and ionization during the electrospray. For analytes with intermediate values of partition coefficient (1.5 < logPow < 4.5) the signals obtained from Fractions B and C were comparable. Overlapping comparison of TIC chromatograms of Fraction B (green line) and of Fraction C (orange line) of a sludge sample fortified with all the analytes at 10 ng/g and internal standard at 100 ng/g is presented in Figure 4. It is noted that all the chromatographic peaks of fractions A, B and C were identified by reference standard solutions prepared in acetonitrile (A, B) and in methanol/water (C), accordingly. It was demonstrated by the reference standard solutions that the solvent did not affect the elution sequence of the analytes but slightly their absolute retention time.

Regarding the comparison between Fraction A and B it was observed that for most analytes eluted at the end of the chromatogram the matrix effect in Fraction A was higher ranging between 28–41%, than the matrix effect observed in Fraction B which ranged between 1–7%. The matrix effect (ME%) was estimated from fortified samples at 10 ng/g according to the following equation ME% = 100 − Rec%. Indicative values are presented in Table S3 (Supplementary Materials). The lower matrix effect of Fraction B can be attributed to the fact that the analytes in this Fraction have been subjected to one additional purification step with PSA comparing to Fraction A (Figure 1) and therefore the extracts of Fraction B contain less matrix components.

It is noted that the common analytes determined in the present work with those determined in the relevant study of agro-food industry sludge from Spain [4] are five out of ten (acetamiprid, carbendazim, imidacloprid, myclobutanil and thiabendazole). There are eight common analytes (azinphos-ethyl, carbendazim, chlorpyrifos, imazalil, imidacloprid, pyriproxyfen, tebuconazole and thiabendazole) out of fifty with the study on the sludge obtained from wastewater treatment plant [5] and four common neonicotinoids pesticides (imidacloprid, clothianidin, thiacloprid and thiamethoxam) out of twelve several analytes of the study in sludge from a pilot plant [6]. It is also highlighted that the sample preparation followed in the present study differs from the others in the material used for the second step of the cleanup with dispersive solid phase extraction which consisted of MgSO_4_ and PSA, while the other studies used other combinations such as MgSO_4_ + PSA + C18 [4,5] or PSA or C18 or PSA + C18 [6]. In addition, the present method considers two fractions (B + C) of the same cleaned-up sample with different solvents, ACN and MeOH/H_2_O (1/1, *v/v*) filtered with different materials, Nylon and PET, respectively before LC injection. Thus, it is revealed that suitable modifications are necessary based on the target analytes of each method.

### 3.3. Method Performance

The SRM chromatograms used for the quantification and identification, SRM1 and SRM2, respectively, of the analytes determined in Fraction C of sludge sample fortified at 10 ng/g, are presented in Figure S1i–vii (Supplementary Materials). The corresponding SRM chromatograms of the analytes determined in Fraction B are presented in Figure S2i–v (Supplementary Materials).

The method performance parameters in terms of limit of detection (LOD), limit of quantification (LOQ), recovery and precision expressed as relative standard deviation (%RSD) at the LOQ, as well as the linear concentration range and the correlation coefficient of the calibration lines are presented in Table 2. The linear regression equations y = a × C+ b along with the *p*-values of the slope (a) and the intercept (b) are presented in Table S4 (Supplementary Materials).

The method LODs ranged between 0.5–5 ng/g for most of the target analytes (41 out of 47), with the majority of the neonicotinoids, the benzimidazole carbendazim, the pyrimidine bupirimate and the pyridine fluopyram to exhibit the highest sensitivity (0.5 ng/g). On the other hand, the organophosphates azinphos ethyl, chlorpyrifos and chlorpyrifos methyl and the pyrethroids lambda cyhalothrin, tau fluvalinate and beta cyfluthrin showed lower sensitivity with LODs between 10–50 ng/g. Accordingly, the method LOQs ranged between 1–10 ng/g for the majority of the target analytes. The LOQ for azinphos ethyl, chlorpyrifos, chlorpyrifos methyl, lambda cyhalothrin and tau fluvalinate was 50 ng/g and for beta cyfluthrin the LOQ was 100 ng/g. It is noteworthy that there is no legislative limit for the monitoring of pesticides in sludge or in soil, however a general data requirement for the limit of quantification in soil in the framework of the European pesticides’ approval is 50 ng/g [22]. The developed method meets this data requirement, except for the case of beta cyfluthrin. In addition, the correlation coefficient of the matrix matched calibration lines is higher than 0.99, for all analytes except for lambda cyhalothrin, beta cyfluthrin and chlorpyrifos. This can be attributed to the increased matrix effect for these substances observed for the sludge samples. The correlation coefficient of the calibration lines prepared in solvent was higher than 0.99 in all cases. The recoveries were in the range of 71–120% and the %RSD was ≤25% for all analytes at the low fortification levels of 1 and 10 ng/g (acceptability criteria 30%) and ≤20% for all the analytes, at fortification levels of 50, 100 and 200 ng/g, except for beta cyfluthrin for which the %RSD was 25% at 100 ng/g. The high values of the RSD could be attributed to the limited homogeneity of the sludge samples.

It is highlighted that the lower validation performance for the pyrethroids is attributed to their chemical structure and physicochemical properties that make them more prone to gas chromatography in comparison to liquid chromatography. Pyrethroids are typically determined by gas chromatography is soil and sludge [3,14,16,23,24] although HPLC analysis was recently reported for the determination of pyrethroids in cereals [25].

### 3.4. Application of the Method

The concentrations of pesticides found in the tested samples and the % moisture of the samples are summarized in Table 3. It can be observed that 37 active substances were quantified in total out of the 47 targeted analytes. The highest concentrations found, expressed in dried weight (dw), concern beta cyfluthrin (3.5 mg/kg dw), tau-fluvalinate (3.2 mg/kg dw), difenoconazole (1.7 mg/kg dw), etofenprox (1.7 mg/kg dw), cyprodinil (1.0 mg/kg dw), phosmet (0.9 mg/kg dw), tebuconazole (0.7 mg/kg dw), fludioxonil (0.7 mg/kg dw), fenoxycarb (0.6 mg/kg dw) and boscalid (0.6 mg/kg dw). It is noted that these compounds have a moderate to high logPow > 2.8. The concentrations of the rest quantified pesticides ranged between 0.005 mg/kg dw (fludioxinil) and 0.434 mg/kg dw (fluopyram). Comparison of these results to the results obtained from agro-food sewage sludge samples from Spain [4] shows good agreement in the common analytes detected and quantified which include acetamiprid, carbendazim, imidacloprid, myclobutanil and thiabendazole.

The lower number of pesticides detected and the lower total pesticide load observed in sludge samples No.1 and No.2 comparing to the other three samples could be attributed to the fact that these samples had lower moisture content (22–24%) and were rather granular with increased particle sizes and thus lower surface/mass ratio, whereas the other samples had high moisture content (72–85%) and were composed from fine-particulate material with high surface/mass ratio and therefore higher adsorbance capacity. However, practically at the end of such a process all the sludge generated from the washing normally ends up in a collection tank and therefore this is the sludge sample that should be considered as regards the total washing effluent management.

## 4. Conclusions

The present work described the development and validation of an LC-ESI (+/−)-MS/MS triple quadrupole method for the determination of 47 pesticides in sludge samples after QuEChERS sample preparation. During the optimization of the method, it was demonstrated that both dispersive solid phase extraction steps of QuEChERS are required for all the target analytes, while the final solvent of the measured solutions was found to significantly affect the sensitivity of analytes with low and high logPow. In addition, the stepwise optimization followed of the LC-ESI-MS/MS parameters, which included, among others, optimization of the drying gas temperature and division of the chromatographic run in segments, led to the development of a reliable, fast, low cost analytical method, using common instrumentation, that can serve for the monitoring of 47 pesticides of different chemical groups, in sludge, with the possibility of adding more active substances in the method. Along with the incorporation of more pesticide active ingredients and their metabolites, other impurities present in the plant protection products and other xenobiotics may be incorporated in this method.

The method was applied to five sludge samples obtained from an agro-food process with the aim to determine the concentration levels of the pesticides in the sludge. This knowledge can be taken into consideration for appropriate handling of this kind of waste at industry scale in a sustainable way. It is noted that although sewage sludge can be characterized as dangerous waste depending on its content in heavy metals according to European legislation [26], currently there is no legislation characterizing sewage sludge as a dangerous waste in terms of its pesticides’ concentration. Although, the Food and Agricultural Organization of the United Nations (FAO) has made available a reference manual for assessing soil contamination by pesticides which aims to help the user determine if pesticide spills have caused soil or groundwater contamination and, if so, whether or not that contamination implies risks for human health [27], further research is required in order to establish standards for the pesticides’ concentration in sludge.

## Figures and Tables

**Table 1:** Selected reaction monitoring (SRM) transitions of the target compounds ordered by retention time (RT). Capillary voltage (CV) and collision energy (CE) for the SRM1 and SRM2.

Analytes	RT (min)	SRM1/SRM2 (*m/z*)	CV (V) SRM1/SRM2	CE (eV) SRM1/SRM2
Flonicamid	3.1	230 > 203/230 > 148	52/52	14/20.5
Carbendazim	3.6	192 > 160/192 > 132	48/48	13/27
Thiamethoxam	3.6	292 > 211/294 > 211	30/30	8.5/20
Thiabendazole	4.4	202 > 175/202 > 131	76/76	19.5/28
Clothianidin	4.6	250 > 169/250 >132	30/30	9.5/11.5
Imidacloprid	4.6	256 > 209/256 >175	40/40	11.5/14
Acetamiprid	5.1	223 > 126/225 > 128	56/56	17/17
Thiacloprid	6.0	253 > 127/255 > 128	48/52	18/17.5
Thiophanate Methyl	7.8	343 > 151/343 > 311	36/36	14.5/7.5
Imazalil	9.3	297 > 159/297 > 201	60/60	18/13.5
Pyrimethanil	9.9	200 > 107/200 > 82	70/70	21/22
Chlorantraniliprole	10.3	484 > 286/484 > 453	36/36	8.5/14
Phosmet	10.4	318 > 160/318 > 133	36/36	8.5/32
Boscalid	10.9	343 > 307/343 > 309	64/64	13.5/14
Cyproconazole	11.2	292 > 70/292 > 125	48/48	13.5/26
Fluxapyroxad	11.2	382 > 362/382 > 342	48/48	9.5/17
Myclobutanil	11.2	289 > 70/289 > 124.9	52/52	12.5/28
Methoxyfenozide	11.3	369 > 149/369 > 313	30/30	13/5.5
Bupirimate	11.5	317 > 166/317 > 108	64/64	20.5/23
Cyprodinil	11.5	226 > 93/226 > 107.9	76/76	30/22
Fluquinconazole	11.5	376 > 307/376 > 349	56/56	23.5/14
Azinphos Ethyl	11.6	346 > 132/346 > 160	30/30	12.5/6
Fluopyram	11.6	397 > 173/397 > 208	60/60	24/16.5
Spirotetramat	11.6	374 > 216/374 > 302	60/60	26.5/12
Fenbuconazole	11.8	337 > 125/337 > 70	64/64	24.5/14
Fenoxycarb	12.0	302 > 116/302 > 88	32/32	7.5/15
Kresoxim Methyl	12.1	314 > 116/314 > 206	30/30	9.5/5
Tebufenozide	12.1	353 > 133/353 > 297	30/30	14.5/5.5
Tebuconazole	12.1	308 > 70/310 > 70	56/60	14.5/14
Triphenyl-Phosphate (I.S)	12.4	327 > 152	88	29
Phosalone	12.5	368 > 182/368 > 111	48/48	10.5/32.5
Pyraclostrobin	12.5	388 > 164/388 > 194	36/36	13.5/8.5
Chlorpyrifos-Methyl	12.6	322 > 125/322 > 290	44/44	15.5/10.5
Difenoconazole	12.7	406 > 251/406 > 337	68/68	20/12
Trifloxystrobin	13.0	409 > 186/409 > 145	40/40	12/39.5
Indoxacarb	13.0	528 > 249/528 > 150	48/48	11.5/20
Pyriproxyfen	13.6	322 > 96/322 > 185	40/40	11.5/19.5
Chlorpyrifos	13.7	352 > 200/350 > 198	36/40	13.5/13.5
Etoxazole	14.1	360 > 141/360 > 113	60/60	27/45
Propargite	14.1	368 > 231/368 > 175	30/30	7/12
Beta Cyfluthrin	14.5	451 > 191/451 > 193	32/32	11.5/11.5
Fenpyroximate	14.5	422 > 135/422 > 107	56/56	28/40.5
Lambda Cyhalothrin	14.7	467 > 466/467 > 225	36/36	5/13.5
Deltamethrin	14.9	524 > 281/524 > 282	36/36	14/13.5
Fluvalinate	15.6	503 > 180/503 > 208	36/36	24.5/8.5
Etofenprox	16.1	394 > 177/394 > 135	30/30	10/20.5
Bifenthrin	16.7	440 > 181/440 > 182	30/30	10.5/10
Fludioxonil ^1^	11.0	247 > 180/247 > 169	68/68	27.5/34.5

^1^ Fludioxonil was monitored in the negative (−) ESI mode.

**Table 2:** Method performance parameters in sludge.

Compound.	LOD (ng/g)	LOQ (ng/g)	%R (LOQ, *n* =5)	%RSD (LOQ, *n* = 5)	Linear Range ng/g	r^2^	FRACTION
Acetamiprid	0.5	1	100	14	1–200	0.9903	C
Bupirimate	0.5	1	108	16	1–200	0.9945	C
Carbendazim	0.5	1	91	21	1–200	0.9939	C
Clothianidin	0.5	1	107	16	1–200	0.9986	C
Fluopyram	0.5	1	77	25	1–200	0.9912	C
Imidacloprid	0.5	1	109	24	1–200	0.9909	C
Thiacloprid	0.5	1	112	18	1–200	0.9934	C
Boscalid	5	10	84	18	10–200	0.9902	C
Chlorantraniliprole	5	10	71	19	10–200	0.9944	C
Cyproconazole	5	10	91	15	10–200	0.9926	C
Cyprodinil	5	10	84	17	10–200	0.9913	C
Difenoconazole	5	10	112	20	10–200	0.9913	C
Fenbuconazole	5	10	84	9	10–200	0.9923	C
Fenoxycarb	5	10	87	8	10–200	0.9923	C
Flonicamid	5	10	76	16	10–200	0.9904	C
Fludioxonil	5	10	89	10	10–200	0.9939	C
Fluquinconazole	5	10	71	8	10–200	0.9921	C
Fluxapyroxad	5	10	104	20	10–200	0.9909	C
Kresoxim Methyl	5	10	84	20	10–200	0.9992	C
Methoxyfenozide	5	10	110	15	10–200	0.9941	C
Myclobutanil	5	10	88	19	10–200	0.9948	C
Tebufenozide	5	10	88	6	10–200	0.9989	C
Tebuconazole	5	10	84	19	10–200	0.9956	C
Thiabendazole	5	10	96	10	10–200	0.9994	C
Thiamethoxam	5	10	90	14	10–200	0.9925	C
Thiophanate Methyl	5	10	82	15	10–200	0.9953	C
Bifenthrin	5	10	98	11	10–200	0.9933	B
Deltamethrin	5	10	98	22	10–200	0.9913	B
Etofenprox	5	10	96	6	10–200	0.9967	B
Etoxazole	5	10	103	13	10–200	0.9916	B
Fenpyroximate	5	10	94	17	10–200	0.9922	B
Imazalil	5	10	108	10	10–200	0.9938	B
Indoxacarb	5	10	85	24	10–200	0.9950	B
Phosalone	5	10	101	15	10–200	0.9946	B
Phosmet	5	10	108	18	10–200	0.9910	B
Propargite	5	10	98	6	10–200	0.9913	B
Pyraclostrobin	5	10	107	13	10–200	0.9961	B
Pyrimethanil	5	10	104	14	10–200	0.9923	B
Pyriproxyfen	5	10	90	19	10–200	0.9935	B
Spirotetramat	5	10	75	7	10–200	0.9918	B
Trifloxystrobin	5	10	111	18	10–200	0.9911	B
Azinphos Ethyl	10	50	91	15	10–200	0.9963	C
Lambda Cyhalothrin	10	50	117	14	50–200	0.9831	B
Chlorpyrifos Methyl	25	50	90	20	50–200	0.9937	B
Chlorpyrifos	25	50	99	21	50–200	0.9862	B
Tau Fluvalinate	25	50	101	20	50–200	0.9960	B
Beta Cyfluthrin	50	100	73	25	50–200	0.9891	B

**Table 3:** Concentrations of pesticides determined in agro-food industry sludge samples.

Analyte	Washing Tank Sample No.1 (mg/Kg dw)	Washing Tank Sample No.2 (mg/Kg dw)	Washing Tank Sample No.3 (mg/Kg dw)	Washing Tank Sample No.4 (mg/Kg dw)	Collection Tank Sample (mg/Kg dw)
Acetamiprid	ND (<0.0005)	ND (<0.0005)	0.078	0.042	0.008
Azinphos Ethyl	ND (<0.01)	ND (<0.01)	ND (<0.01)	ND (<0.01)	ND (<0.01)
Beta Cyfluthrin	0.097	0.027	3.56	3.56	3.425
Bifenthrin	ND (<0.005)	ND (<0.005)	0.015	0.025	0.080
Boscalid	ND (<0.005)	ND (<0.005)	0.590	0.045	0.088
Bupirimate	ND (<0.0005)	ND (<0.0005)	ND (<0.0005)	ND (<0.0005)	ND (<0.0005)
Carbendazim	ND (<0.0005)	ND (<0.0005)	0.023	0.021	0.011
Chlorantraniliprole	ND (<0.005)	ND (<0.005)	0.399	0.126	0.068
Chlorpyrifos-Methyl	ND (<0.025)	ND (<0.025)	ND (<0.025)	ND (<0.025)	ND (<0.025)
Chlorpyrifos-Ethyl	ND (<0.025)	ND (<0.025)	0.101	0.181	0.075
Clothianidin	ND (<0.005)	ND (<0.005)	0.011	0.022	ND (<0.005)
Cyproconazole	ND (<0.005)	ND (<0.005)	ND (<0.005)	ND (<0.005)	ND (<0.005)
Cyprodinil	ND (<0.005)	ND (<0.005)	1.03	0.50	0.119
Deltamethrin	ND (<0.005)	ND (<0.005)	0.171	0.137	0.047
Difenoconazole	0.010	0.010	1.69	0.38	0.672
Etofenprox	ND (<0.005)	ND (<0.005)	1.73	0.12	0.062
Etoxazole	ND (<0.005)	ND (<0.005)	0.066	0.011	0.026
Fenbuconazole	ND (<0.005)	ND (<0.005)	ND (<0.005)	ND (<0.005)	0.010
Fenoxycarb	ND (<0.005)	ND (<0.005)	0.575	0.210	0.043
Fenpyroximate	ND (<0.005)	ND (<0.005)	ND (<0.005)	ND (<0.005)	ND (<0.005)
Flonicamid	ND (<0.005)	ND (<0.005)	ND (<0.005)	ND (<0.005)	ND (<0.005)
Fludioxonil	0.005	0.005	0.680	0.095	0.208
Fluopyram	ND (<0.0005)	ND (<0.0005)	0.090	0.434	0.012
Fluquinconazole	ND (<0.005)	ND (<0.005)	ND (<0.005)	0.021	0.012
Tau-Fluvalinate	0.045	ND (<0.025)	3.17	2.05	0.706
Fluxapyroxad	ND (<0.005)	ND (<0.005)	0.087	0.072	0.021
Imazalil	ND (<0.005)	ND (<0.005)	0.198	0.104	0.165
Imidacloprid	ND (<0.0005)	ND (<0.0005)	ND (<0.0005)	0.007	ND (<0.0005)
Indoxacarb	ND (<0.005)	ND (<0.005)	0.362	0.120	0.096
Kresoxim Methyl	ND (<0.005)	ND (<0.005)	ND (<0.005)	ND (<0.005)	ND (<0.005)
Lambda Cyhalothrin	0.015	ND (<0.01)	0.471	0.305	0.338
Methoxyfenozide	ND (<0.005)	ND (<0.005)	0.240	0.041	0.037
Myclobutanil	ND (<0.005)	0.013	0.294	0.198	0.043
Phosalone	ND (<0.005)	ND (<0.005)	ND (<0.005)	ND (<0.005)	ND (<0.005)
Phosmet	ND (<0.005)	ND (<0.005)	0.906	0.114	0.012
Propargite	ND (<0.005)	ND (<0.005)	0.019	0.034	ND (<0.005)
Pyraclostrobin	ND (<0.005)	ND (<0.005)	0.059	0.023	0.005
Pyrimethanil	ND (<0.005)	ND (<0.005)	0.010	0.005	ND (<0.005)
Pyriproxyfen	ND (<0.005)	ND (<0.005)	0.087	0.090	ND (<0.005)
Spirotetramat	ND (<0.005)	ND (<0.005)	ND (<0.005)	ND (<0.005)	ND (<0.005)
Tebufenozide	ND (<0.005)	ND (<0.005)	0.354	0.099	0.132
Tebuconazole	ND (<0.005)	ND (<0.005)	0.752	0.312	0.111
Thiacloprid	ND (<0.0005)	0.002	0.243	0.385	0.063
Thiabendazole	0.007	0.005	0.571	0.218	0.505
Thiamethoxam	ND (<0.005)	ND (<0.005)	ND (<0.005)	0.099	ND (<0.005)
Thiophanate Methyl	ND (<0.005)	ND (<0.005)	ND (<0.005)	ND (<0.005)	ND (<0.005)
Trifloxystrobin	ND (<0.005)	ND (<0.005)	0.309	0.171	0.010
Total pesticide load per sludge sample (mg/Kg dw)	0.2	0.1	19.0	10.4	7.2
Number of pesticides detected per sludge sample	6	6	33	36	31
Moisture %	24	22	85	73	72

## Data Availability

Not applicable.

## Supplementary Materials

The following are available online, Table S1: Target compounds’ molecular weight, formula and structure (ordered by retention time, RT), function and partition coefficient pKow (pH 7, 20 °C), Table S2: Number of SRMs included in each time-segment, dwell time of each transition, total scan time of each time-segment and the drying gas temperature set for each time-segment, Table S3: Matrix effect on the signal of late eluting analytes obtained in Fraction A and B (RT: Retention time), Table S4: Linear regression equations, y = a × C+ b, of the matrix-matched curves where y is the ratio of the Peak area of the analyte/Peak area of the internal standard and C, the concentration in ng/g, Figure S1: (i). Quantification (SRM1) and confirmation (SRM2) chromatograms of analytes flonicamid-clothianidin determined in Fraction C of sludge sample fortified at 10 ng/g, (ii). Quantification (SRM1) and confirmation (SRM2) chromatograms of analytes imidacloprid-chloratranilipole determined in Fraction C of sludge sample fortified at 10 ng/g, (iii). Quantification (SRM1) and confirmation (SRM2) chromatograms of analytes boscalid-methoxyfenozide determined in Fraction C of sludge sample fortified at 10 ng/g, (iv). Quantification (SRM1) and confirmation (SRM2) chromatograms of analytes bupirimate-fluopyram determined in Fraction C of sludge sample fortified at 10 ng/g, (v). Quantification (SRM1) and confirmation (SRM2) chromatograms of analytes fenbuconazole-tebuconazole determined in Fraction C of sludge sample fortified at 10 ng/g, (vi). Quantification (SRM1) and confirmation (SRM2) chromatograms of analytes triphenyl-phosphate (I.S)-difenoconazole determined in Fraction C of sludge sample fortified at 10 ng/g, (vii). Quantification (SRM1) and confirmation (SRM2) chromatograms of analyte Fludioxonil determined in Fraction C of sludge sample fortified at 10 ng/g, in negative polarity, Figure S2: (i). Quantification (SRM1) and Confirmation (SRM2) chromatograms of analytes imazalil-triphenyl-phosphate (I.S) determined in Fraction B of sludge sample fortified at 10 ng/g, (ii). Quantification (SRM1) and confirmation (SRM2) chromatograms of analytes phosalone-indoxacarb determined in Fraction B of sludge sample fortified at 10 ng/g, (iii). Quantification (SRM1) and confirmation (SRM2) chromatograms of analytes pyriproxyfen-b-cyfluthrin determined in Fraction B of sludge sample fortified at 10–200 ng/g, (iv). Quantification (SRM1) and confirmation (SRM2) chromatograms of analytes fenpyroximate-etofenprox determined in Fraction B of sludge sample fortified at 10–50 ng/g, (v). Quantification (SRM1) and confirmation (SRM2) chromatograms of analyte bifenthrin determined in Fraction B of sludge sample fortified at 10 ng/g.

## Sample Availability

Samples of the compounds are not available from the authors.
