# Supplementary material for: Targeted Multiresidue Method for the Analysis of Different Classes of Pesticides in Agro-Food Industrial Sludge by Liquid Chromatography Tandem Mass Spectrometry
Source: Molecules. 2021 Nov 15;26(22):6888. doi: 10.3390/molecules26226888 (PMC8617938; doi:10.3390/molecules26226888)

Targeted multiresidue method for the analysis of different classes of pesticides in agro-food industrial sludge by liquid chromatography tandem mass spectrometry

Niki C. Maragou [1,*], George Balayiannis [1], Evangelos Karanasios [2] , Emilia Markellou [3] and Konstantinos Liapis [4]

[1] *Laboratory of Chemical Control of Pesticides, Scientific Directorate of Pesticides' Control and Phytopharmacy, Benaki Phytopathological Institute, 8 St. Delta Street, Kifissia, 14561 Athens, Greece*; n.maragou@bpi.gr (N.C.M.); g.balayiannis@bpi.gr (G.B)
[2] Laboratory of Environmental Control of Pesticides, Scientific Directorate of Pesticides' Control & Phytopharmacy, Benaki Phytopathological Institute, 8 St. Delta Street, Kifissia, 14561 Athens, Greece; e.karanasios@bpi.gr
[3] Laboratory of Mycology, Scientific Directorate of Phytopathology, Benaki Phytopathological Institute, 8 St. Delta Street, Kifissia, 14561 Athens, Greece; e.markellou@bpi.gr
[4] Laboratory of Pesticide Residues, Scientific Directorate of Pesticides' Control and Phytopharmacy, Benaki Phytopathological Institute, 8 St. Delta Street, Kifissia, 14561 Athens, Greece; k.liapis@bpi.gr

* Correspondence: n.maragou@bpi.gr; Tel.: (+302108180350)

Supplementary material

Table S1. Target compounds' molecular weight, formula and structure (ordered by retention time, RT), function and partition coefficient pKow (pH 7, 20 °C).

Analytes	Molecular Formula	MW (g/mol)	Structure	Chemical class [18,19] (Function)	pKow pH 7, 20 °C [18]	RT (min)
Flonicamid	$C_9H_6F_3N_3O$	229.2	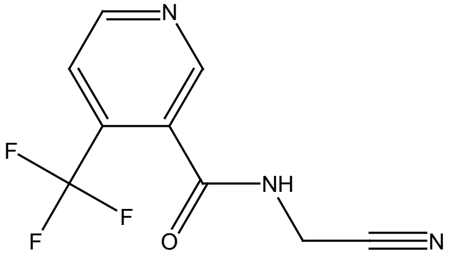	pyridinecarboxamide (Insecticide; aphicide)	-0.24	3.1
Carbendazim	$C_9H_9N_3O_2$	191.2	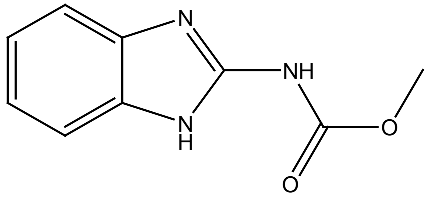	benzimidazole benzimidazolylcarbamate (fungicide; metabolite of thiophanate methyl)	1.48	3.6
Thiamethoxam	$C_8H_{10}ClN_5O_3S$	291.7	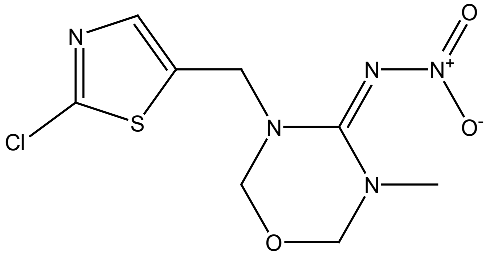	neonicotinoid & thiazole (Insecticide)	-0.13	3.6
Thiabendazole	$C_{10}H_7N_3S$	201.3	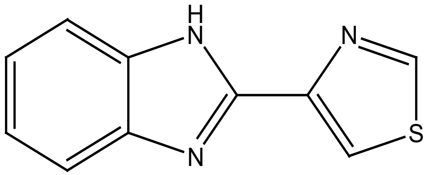	benzimidazole & thiazole (Fungicide)	2.39	4.4

Analytes	Molecular Formula	MW (g/mol)	Structure	Chemical class [18,19] (Function)	pKow pH 7, 20 °C [18]	RT (min)
Clothianidin	$C_6H_8ClN_5O_2S$		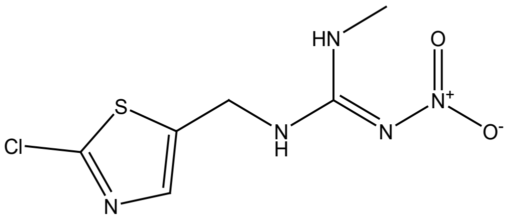	neonicotinoid<br>&<br>thiazole<br>(Insecticide; metabolite of thiamethoxam)	0.905	4.6
Imidacloprid	$C_9H_{10}ClN_5O_2$	255.6	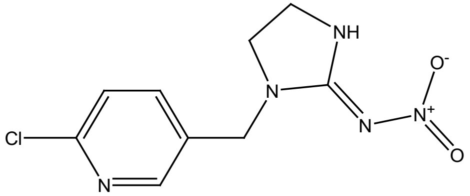	neonicotinoid<br>(Insecticide)	0.57	4.6
Acetamiprid	$C_{10}H_{11}ClN_4$	222.7	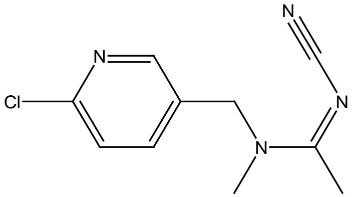	neonicotinoid<br>(Insecticide)	0.8	5.1
Thiacloprid	$C_{10}H_9ClN_4S$	252.7	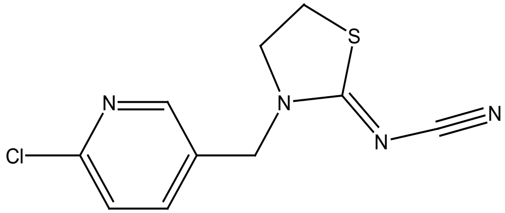	neonicotinoid<br>&<br>thiazolidine<br>(Insecticide)	1.26	6.0

Analytes	Molecular Formula	MW (g/mol)	Structure	Chemical class [18,19] (Function)	pKow pH 7, 20 °C [18]	RT (min)
Thiophanate Methyl	$C_{12}H_{14}N_4O_4S_2$	342.4	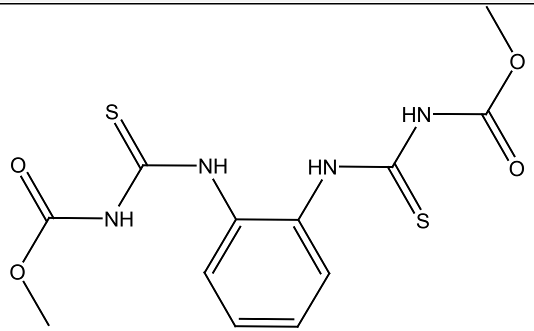	benzimidazole precursor & carbamate (Fungicide)	1.4	7.8
Imazalil	$C_{14}H_{14}Cl_2N_2O$	297.2	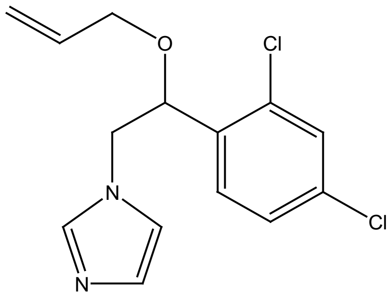	conazole /imidazoles (Fungicide)	2.56	9.3
Pyrimethanil	$C_{12}H_{13}N_3$	199.3	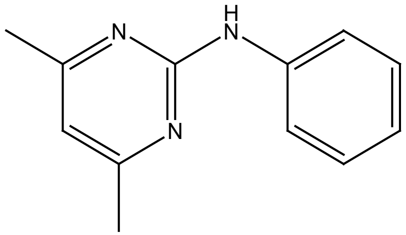	anilinopyrimidine (Fungicide)	2.84	9.9

Analytes	Molecular Formula	MW (g/mol)	Structure	Chemical class [18,19] (Function)	pKow pH 7, 20 °C [18]	RT (min)
Chlorantraniliprole	$C_{18}H_{14}BrCl_2N_5O_2$		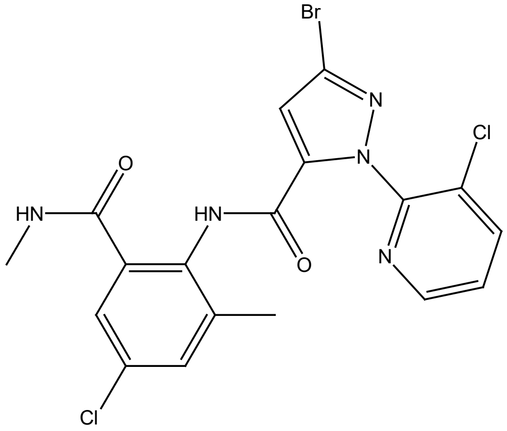	diamide & pyridylpyrazole (insecticide)	2.86	10.3
Phosmet	$C_{11}H_{12}NO_4PS_2$	317.3	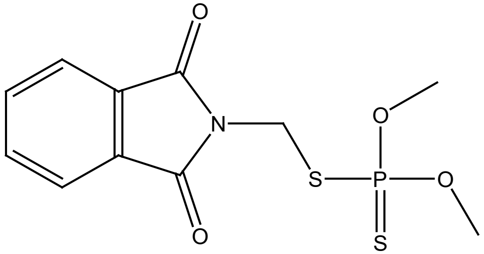	organothiophosphate/ isoindole & phthalimide (Insecticide; acaricide)	2.8	10.4
Boscalid	$C_{18}H_{12}Cl_2N_2O$	343.2	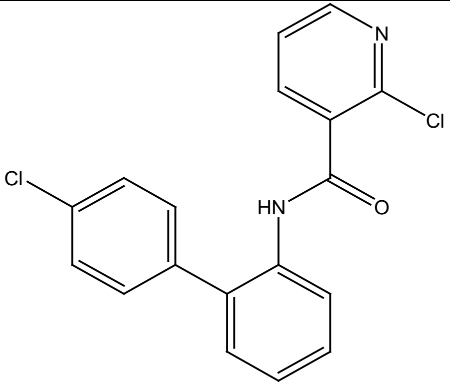	anilide & pyridine (Fungicide)	2.96	10.9

Analytes	Molecular Formula	MW (g/mol)	Structure	Chemical class [18,19] (Function)	pKow pH 7, 20 °C [18]	RT (min)
Cyproconazole	$C_{15}H_{18}ClN_3O$		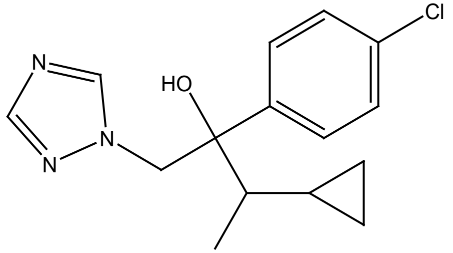	conazole / triazoles (Fungicide)	3.09	11.2
Fluxapyroxad	$C_{18}H_{12}F_5N_3O$	381.31	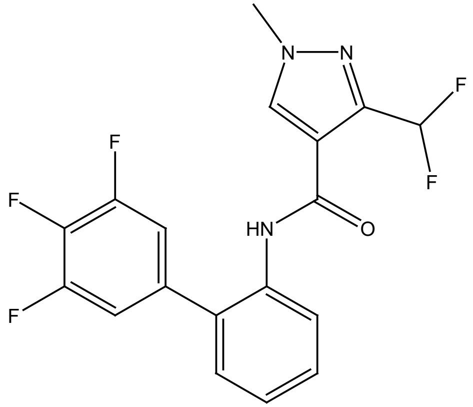	anilide & pyrazolecarboxamide (Fungicide)	3.13	11.2
Myclobutanil	$C_{15}H_{17}ClN_4$	288.78	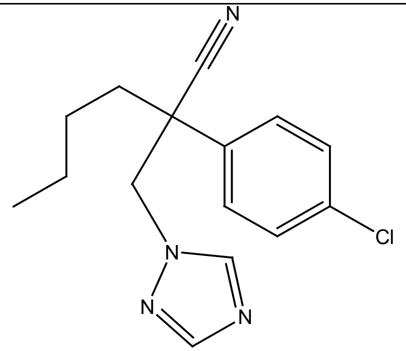	conazole / triazoles (Fungicide)	2.89	11.2

Analytes	Molecular Formula	MW (g/mol)	Structure	Chemical class [18,19] (Function)	pKow pH 7, 20 °C [18]	RT (min)
Methoxyfenozide	$C_{22}H_{28}N_2O_3$	368.5	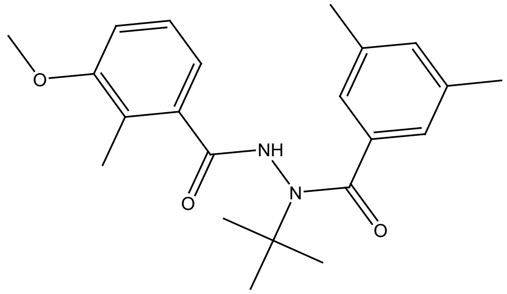	moulting hormone agonists (insecticide)	3.72	11.3
Bupirimate	$C_{13}H_{24}N_4O_3S$	316.5	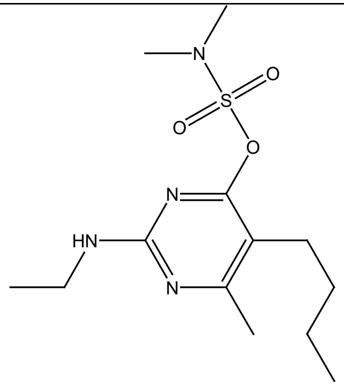	pyrimidine (Fungicide)	3.68	11.5
Cyprodinil	$C_{14}H_{15}N_3$	225.3	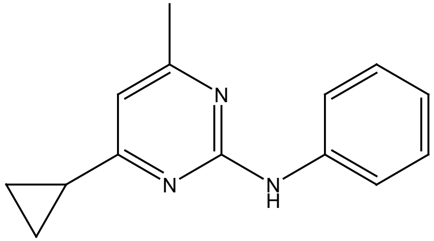	pyrimidine (Fungicide)	4	11.5

Analytes	Molecular Formula	MW (g/mol)	Structure	Chemical class [18,19] (Function)	pKow pH 7, 20 °C [18]	RT (min)
Fluquinconazole	$C_{16}H_8Cl_2FN_5O$	376.2	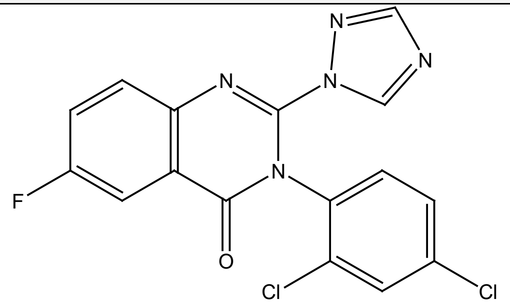	conazole / triazoles (Fungicide)	3.24	11.5
Azinphos Ethyl	$C_{12}H_{16}N_3O_3PS_2$		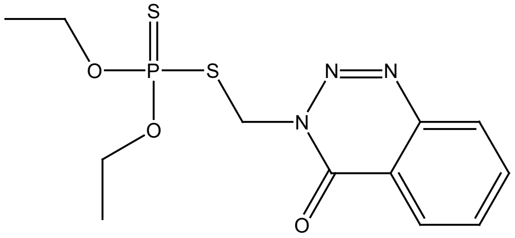	benzotriazine organothiophosphate (acaricide; insecticide)	3.18	11.6
Fluopyram	$C_{16}H_{11}ClF_6N_2O$		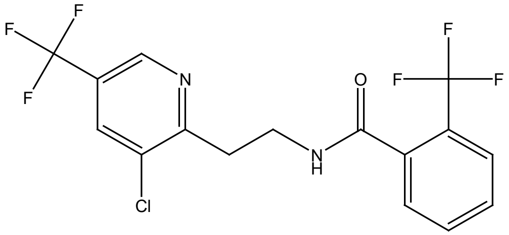	Benzamide & pyridine (fungicide)	3.3	11.6

Analytes	Molecular Formula	MW (g/mol)	Structure	Chemical class [18,19] (Function)	pKow pH 7, 20 °C [18]	RT (min)
Spirotetramat	$C_{21}H_{27}NO_5$		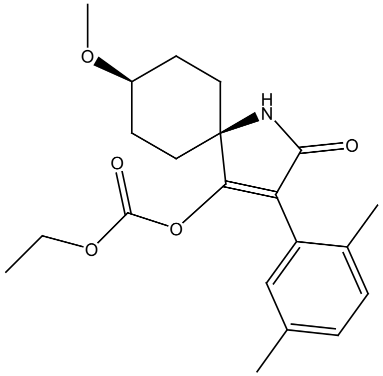	tetramic acid (insecticide)	2.51	11.6
Fenbuconazole	$C_{19}H_{17}ClN_4$	336.82	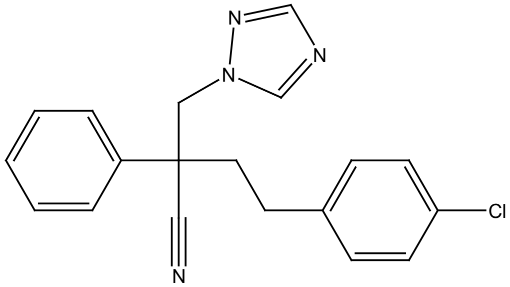	conazole / triazoles (fungicide)	3.79	11.8
Fenoxycarb	$C_{17}H_{19}NO_4$	301.4	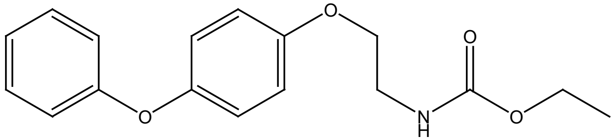	juvenile hormone mimics (insecticide)	4.07	12.0
Kresoxim Methyl	$C_{18}H_{19}NO_4$	313.35	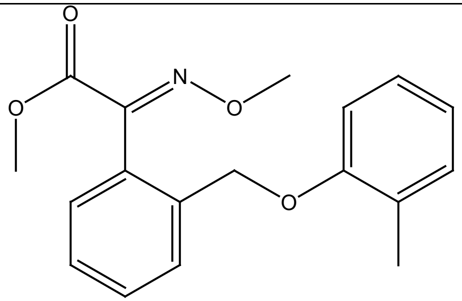	methoxyiminoacetate strobilurin (fungicide)	3.4	12.1

Analytes	Molecular Formula	MW (g/mol)	Structure	Chemical class [18,19] (Function)	pKow pH 7, 20 °C [18]	RT (min)
Tebuconazole	$C_{16}H_{22}ClN_3O$	307.8	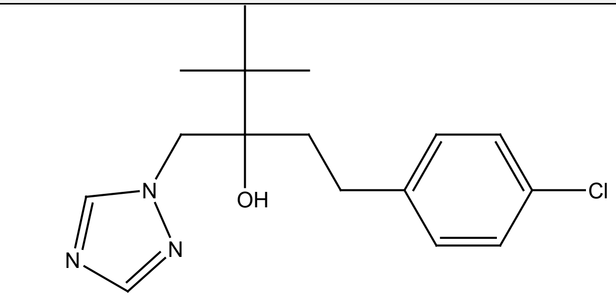	conazole / triazoles (fungicide)	3.7	12.1
Tebufenozide	$C_{22}H_{28}N_2O_2$	352.47	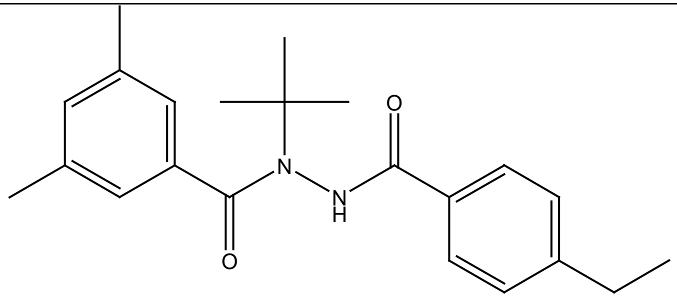	moulting hormone agonists (insecticide)	4.25	12.1
Phosalone	$C_{12}H_{15}ClNO_4PS_2$	367.8	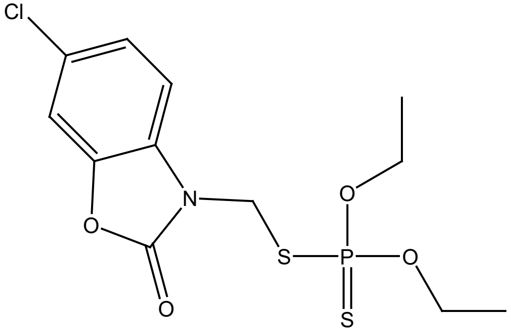	heterocyclic organothiophosphate (insecticide; acaricide)	4.01	12.5

Analytes	Molecular Formula	MW (g/mol)	Structure	Chemical class [18,19] (Function)	pKow pH 7, 20 °C [18]	RT (min)
Pyraclostrobin	$C_{19}H_{18}ClN_3O_4$	387.8	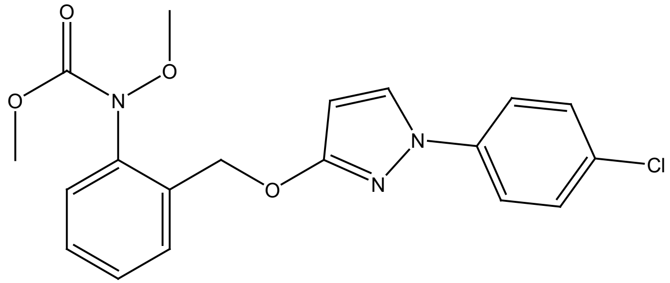	phenylpyrazole & methoxycarbanilate strobilurin (fungicide)	3.99	12.5
Chlorpyrifos-Methyl	$C_7H_7Cl_3NO_3PS$	322.53	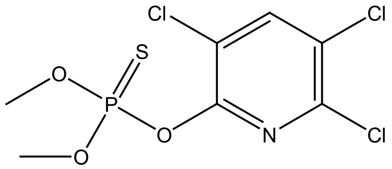	pyridine organothiophosphate (insecticide)	4.00	12.6
Difenoconazole	$C_{19}H_{17}Cl_2N_3O_3$	406.26	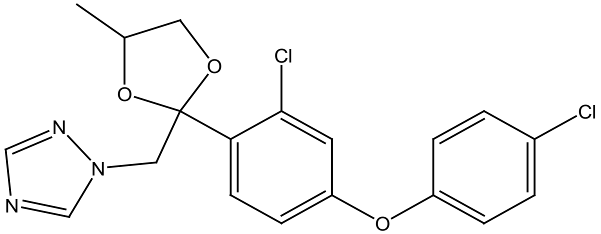	conazole / triazoles (fungicide)	4.36	12.7
Trifloxystrobin	$C_{20}H_{19}F_3N_2O_4$	408.4	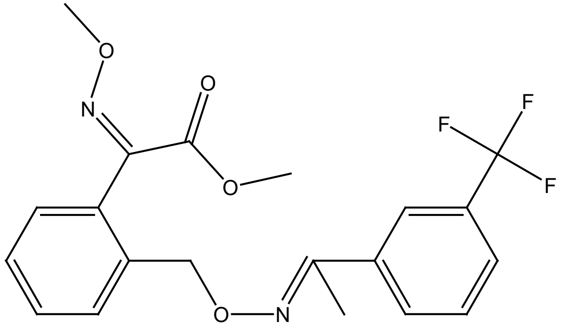	methoxyiminoacetate strobilurin (fungicide)	4.5	13.0

Analytes	Molecular Formula	MW (g/mol)	Structure	Chemical class [18,19] (Function)	pKow pH 7, 20 °C [18]	RT (min)
Indoxacarb	$C_{22}H_{17}ClF_3N_3O_7$	527.83	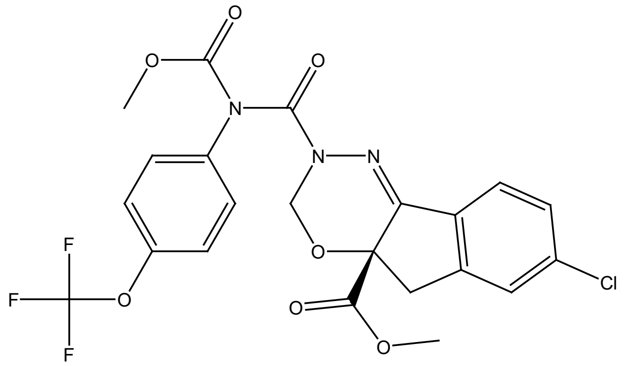	oxadiazine (insecticide)	4.65	13.0
Pyriproxyfen	$C_{20}H_{19}NO_3$	321.4	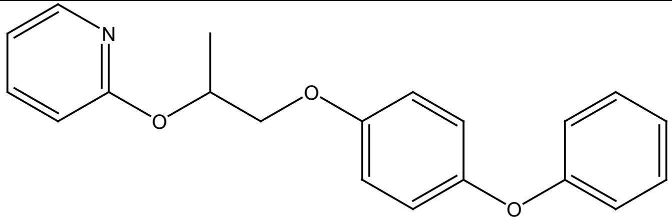	juvenile hormone mimics (insecticide)	5.37	13.6
Chlorpyrifos	$C_9H_{11}Cl_3NO_3PS$	350.58	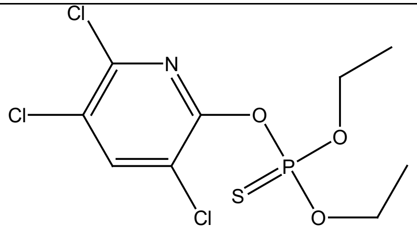	pyridine organothiophosphate (acaricide; insecticide)	4.7	13.7
Etoxazole	$C_{21}H_{23}F_2NO_2$	359.42	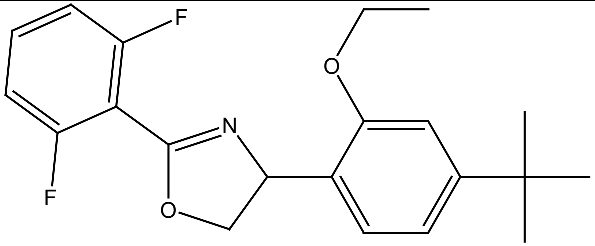	diphenyl oxazoline (acaricide)	5.52	14.1

Analytes	Molecular Formula	MW (g/mol)	Structure	Chemical class [18,19] (Function)	pKow pH 7, 20 °C [18]	RT (min)
Propargite	$C_{19}H_{26}O_4S$	350.5	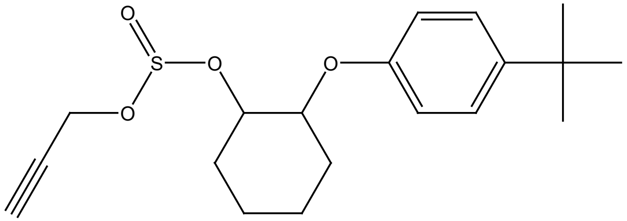	sulfite ester (acaricide)	5.7	14.1
Beta Cyfluthrin	$C_{22}H_{18}Cl_2FNO_3$	434.3	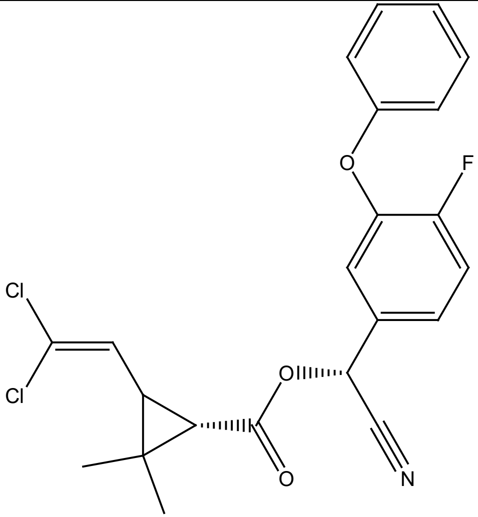	pyrethroid ester (insecticide)	5.85	14.5
Fenpyroximate	$C_{24}H_{27}N_3O_4$	421.49	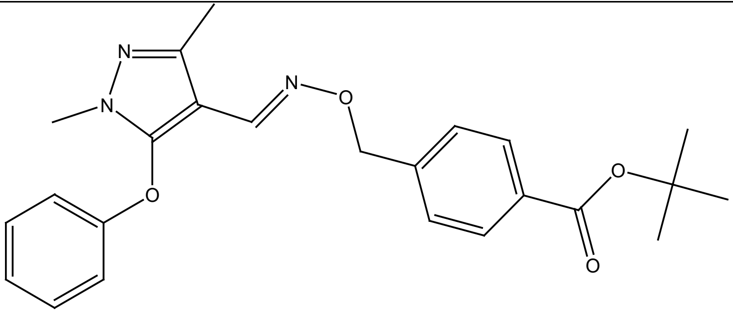	pyrazole (acaricide)	5.70	14.5

Analytes	Molecular Formula	MW	Structure	Chemical class [18,19] (Function)	pKow pH 7, 20 °C [18]	RT (min)
Lambda Cyhalothrin	$C_{23}H_{19}ClF_3NO_3$	449.9	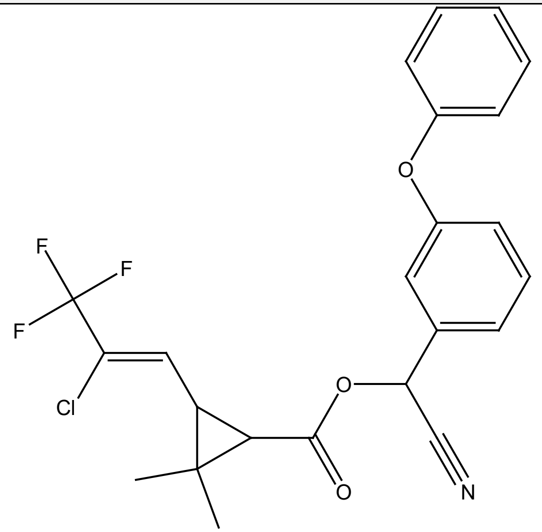	pyrethroid ester (insecticide)	5.5	14.7
Deltamethrin	$C_{22}H_{19}Br_2NO_3$	505.2	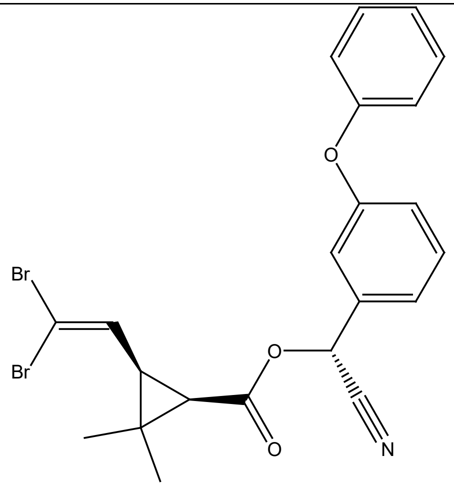	pyrethroid ester (insecticide)	4.6	14.9

Analytes	Molecular Formula	MW (g/mol)	Structure	Chemical class [18,19] (Function)	pKow pH 7, 20 °C [18]	RT (min)
Tau-fluvalinate	$C_{26}H_{22}ClF_3N_2O_3$	502.9	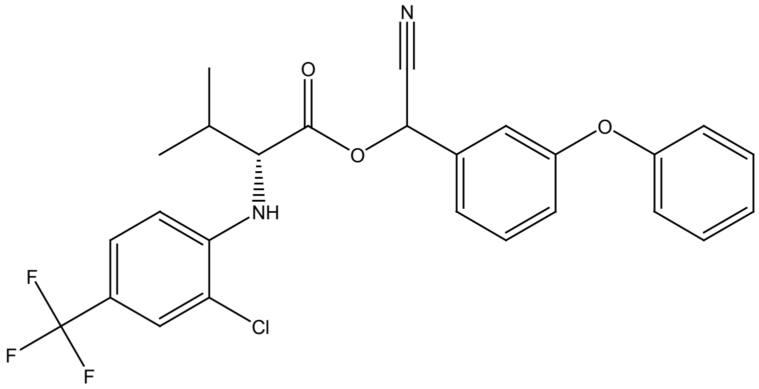	pyrethroid ester (acaricide; insecticide)	7.02	15.6
Etofenprox	$C_{25}H_{28}O_3$	376.49	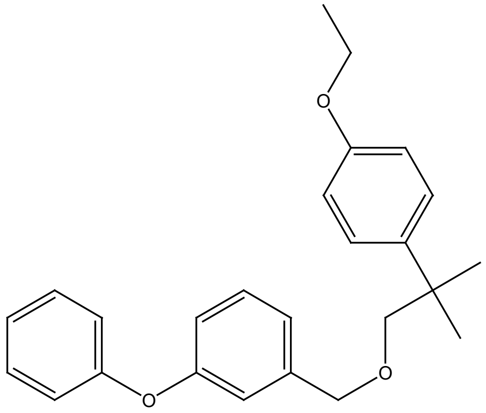	pyrethroid ether (insecticide)	6.9	16.1

Analytes	Molecular Formula	MW (g/mol)	Structure	Chemical class [18,19] (Function)	pKow pH 7, 20 °C [18]	RT (min)
Bifenthrin	$C_{23}H_{22}ClF_3O_2$	422.88	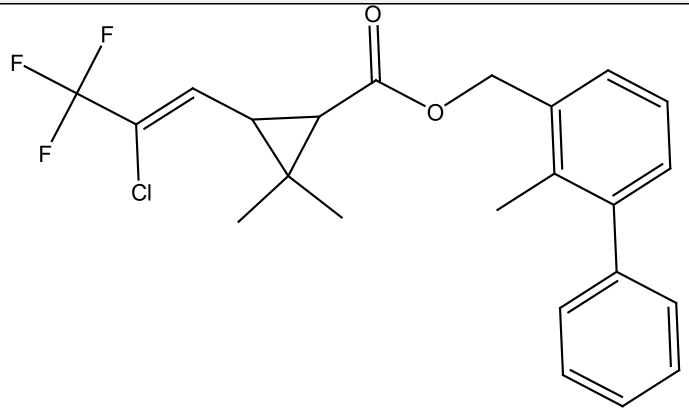	pyrethroid ester (acaricide; insecticide)	6.6	16.7
Fludioxonil [1]	$C_{12}H_6F_2N_2O_2$	248.2	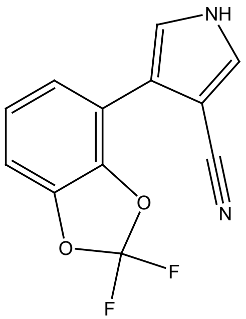	pyrrole (fungicide)	4.12	11.0

[1] Fludioxonil was monitored in the negative (-) ESI mode.

Table S2. Number of SRMs included in each time-segment, dwell time of each transition, total scan time of each time-segment and the drying gas temperature set for each time-segment.

Segment	Number of SRMs	Dwell time (sec)	Scan time (sec)	Drying gas T (°C)
1	1	0.100	0.10	340
2	1	0.100	0.10	340
3	4	0.250	1.00	340
4	12	0.100	1.20	330
5	14	0.100	1.40	320
6	12	0.100	1.20	310
7	6	0.150	0.90	300
8	4	0.250	1.00	290
9	4	0.250	1.00	280
10	10	0.100	1.00	270
11	30	0.030 - 0.050	1.42	260
12	54	0.025 - 0.030	1.61	250
13	54	0.025	1.35	240
14	50	0.025	1.25	230
15	26	0.025 - 0.050	1.05	220
16	22	0.050 - 0.100	1.30	210
17	10	0.100	1.00	200
18	4	0.250	1.00	200
19	1	0.100	0.10	200
20	1	0.100	0.10	200

Table S3. Matrix effect on the signal of late eluting analytes obtained in Fraction A and B (RT: Retention time)

	RT (min)	Matrix Effect Fraction A	Matrix Effect Fraction B
**ETOXAZOLE**	14.1	30	-4
**PROPARGITE**	14.1	28	-7
**FENPYROXIMATE**	14.5	32	6
**ETOFENPROX**	16.1	30	-4
**BIFENTHRIN**	16.7	41	-4

Table S4. Linear regression equations, y = a × C+ b, of the matrix-matched curves where y is the ratio of the Peak area of the analyte/Peak area of the internal standard and C, the concentration in ng/g.

Compound	Linear range ng/g	y = a × C+ b	p-value slope (a)	p-value intercept (b)	r^2
Acetamiprid	1 – 200	y = 0.0119 × C + 0.0797	2.4E-19	0.0169	0.9903
Bupirimate	1 – 200	y = 0.0197 × C + 0.0760	3.8E-21	0.0356	0.9945
Carbendazim	1 – 200	y = 0.0221 × C + 0.1125	3.0E-18	0.0913	0.9939
Clothianidin	1 – 200	y = 0.0018 × C + 0.014	1.2E-17	0.0024	0.9986
Fluopyram	1 – 200	y = 0.0169 × C + 0.054	1.1E-22	0.0832	0.9912
Imidacloprid	1 – 200	y = 0.0028 × C + 0.017	4.6E-19	0.0138	0.9909
Thiacloprid	1 – 200	y = 0.0190 × C + 0.1334	1.3E-18	0.0209	0.9934
Boscalid	10 – 200	y = 0.0055 × C + 0.0541	2.6E-14	0.0109	0.9902
Chlorantraniliprole	10 – 200	y = 0.0030 × C + 0.0162	2.6E-16	0.0403	0.9944
Cyproconazole	10 – 200	y = 0.0084 × C + 0.0729	2.3E-13	0.0171	0.9926
Cyprodinil	10 – 200	y = 0.0084 × C + 0.0782	1.9E-12	0.0096	0.9913
Difenoconazole	10 – 200	y = 0.0130 × C + 0.1297	1.2E-13	0.0188	0.9913
Fenbuconazole	10 – 200	y = 0.0048 × C + 0.0607	3.7E-13	0.0019	0.9929
Fenoxycarb	10 – 200	y = 0.0098 × C + 0.1087	3.8E-15	0.0004	0.9923
Flonicamid	10 – 200	y = 0.0006 × C + 0.0082	3.0E-11	0.0039	0.9904
Fludioxonil*	10 – 200	y = 291840× C + 102051	7.8E-14	0.9067	0.9939
Fluquinconazole	10 – 200	y = 0.0006 × C + 0.0058	1.4E-13	0.0059	0.9921
Fluxapyroxad	10 – 200	y = 0.0094 × C + 0.0612	6.1E-15	0.0032	0.9909

Kresoxim Methyl	10 – 200	y = 0.0016 × C + 0.0159	1.3E-12	0.0185	0.9992
Methoxyfenozide	10 – 200	y = 0.0152 × C + 0.0629	6.1E-15	0.1950	0.9941
Myclobutanil	10 – 200	y = 0.0075 × C + 0.0652	9.3E-14	0.0120	0.9948
Tebufenozide	10 – 200	y = 0.0120 × C + 0.0607	3.1E-17	0.0008	0.9989
Tebuconazole	10 – 200	y = 0.0079 × C + 0.0752	2.9E-16	0.0017	0.9956
Thiabendazole	10 – 200	y = 0.0082 × C + 0.0967	1.2E-12	0.0196	0.9994
Thiamethoxam	10 – 200	y = 0.0017 × C + 0.0193	2.8E-14	0.0044	0.9925
Thiophanate Methyl	10 – 200	y = 0.0242 × C + 0.3306	2.3E-11	0.0039	0.9953
Bifenthrin	10 – 200	y = 0.0051 × C + 0.0285	1.2E-11	0.0963	0.9933
Deltamethrin	10 – 200	y = 0.0016 × C + 0.0113	7.0E-14	0.0714	0.9913
Etofenprox	10 – 200	y = 0.0296 × C + 0.1262	1.5E-12	0.1160	0.9967
Etoxazole	10 – 200	y = 0.0820 × C + 0.5444	7.3E-15	0.0490	0.9916
Fenpyroximate	10 – 200	y = 0.0074 × C + 0.0520	2.7E-15	0.0159	0.9922
Imazalil	10 – 200	y = 0.0064 × C + 0.0645	2.3E-15	0.0030	0.9938
Indoxacarb	10 – 200	y = 0.0017 × C + 0.0157	4.8E-12	0.0633	0.9950
Phosalone	10 – 200	y = 0.0117 × C + 0.0615	9.8E-14	0.1785	0.9946
Phosmet	10 – 200	y = 0.0134 × C + 0.1199	1.6E-14	0.0157	0.9910
Propargite	10 – 200	y = 0.0166 × C + 0.0706	1.0E-16	0.0301	0.9913
Pyraclostrobin	10 – 200	y = 0.0223 × C + 0.1271	1.2E-15	0.0535	0.9961
Pyrimethanil	10 – 200	y = 0.0057 × C + 0.0578	7.3E-14	0.0149	0.9923
Pyriproxyfen	10 – 200	y = 0.0482 × C + 0.2375	4.0E-13	0.0853	0.9935
Spirotetramat	10 – 200	y = 0.0075 × C + 0.0532	5.8E-15	0.0336	0.9918
Trifloxystrobin	10 – 200	y = 0.0326 × C + 0.1391	1.9E-13	0.1961	0.9911

Azinphos Ethyl	10 – 200	y = 0.0008 × C + 0.0038	2.1E-14	0.1673	0.9963
Lambda Cyhalothrin	50 – 200	y = 0.0027 × C + 0.0128	1.8E-07	0.4071	0.9831
Chlorpyrifos Methyl	50 – 200	y = 0.0006 × C + 0.0043	1.0E-11	0.1849	0.9937
Chlorpyrifos	50 – 200	y = 0.0011 × C + 0.0057	7.6E-09	0.1817	0.9862
Tau Fluvalinate	50 – 200	y = 0.0047 × C + 0.0007	1.1E-09	0.9667	0.9960
Beta Cyfluthrin	50 – 200	y = 0.0001 × C + 0.0078	2.5E-04	0.0249	0.9891

* Measured in negative ESI without internal standard (y corresponds to the peak area)

Figure S1 (i). Quantification (SRM1) and Confirmation (SRM2) chromatograms of analytes Flonicamid – Clothianidin determined in Fraction C of sludge sample fortified at 10 ng/g.

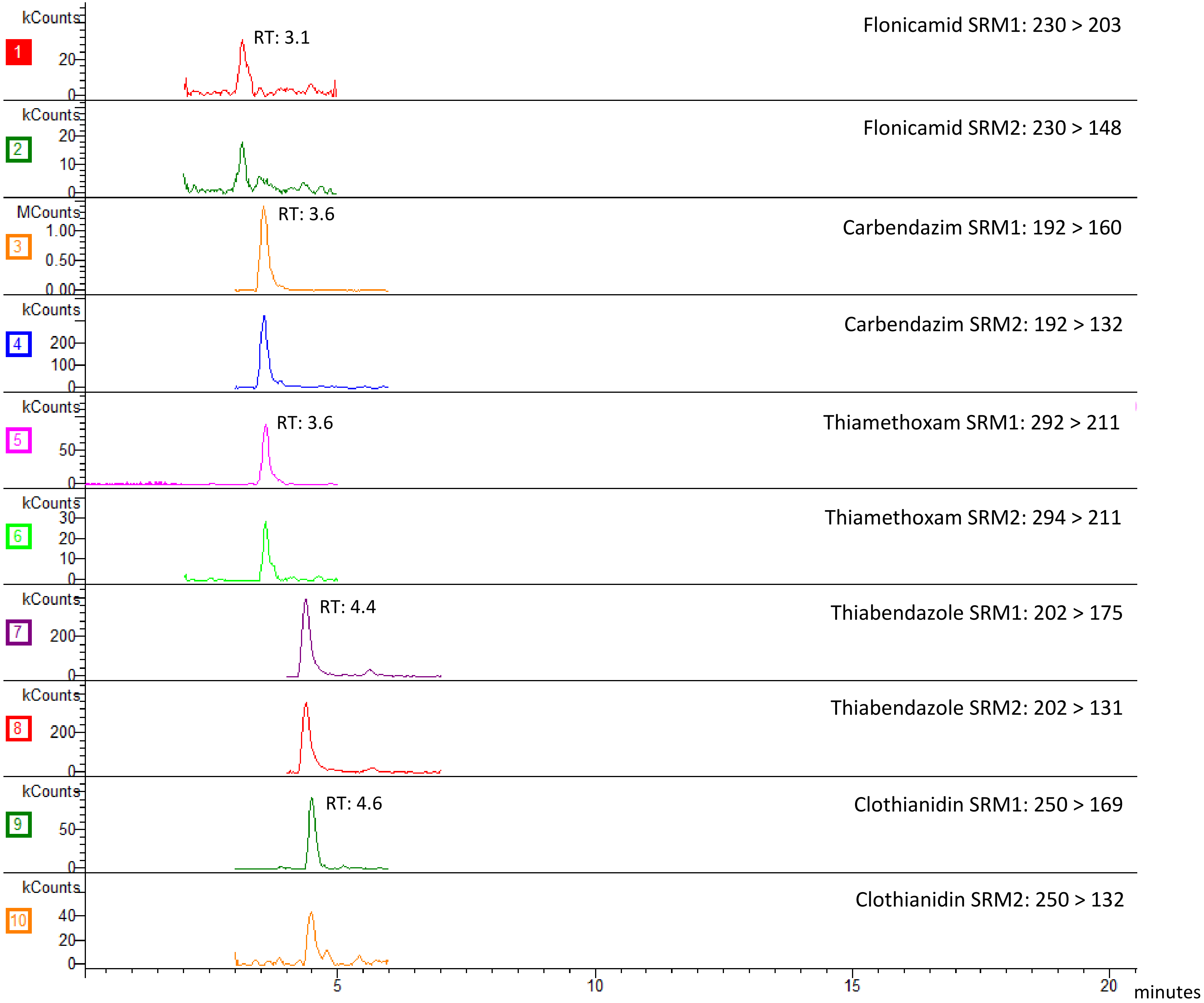

Figure S1 (ii). Quantification (SRM1) and Confirmation (SRM2) chromatograms of analytes Imidacloprid – Chloratranilipole determined in Fraction C of sludge sample fortified at 10 ng/g.

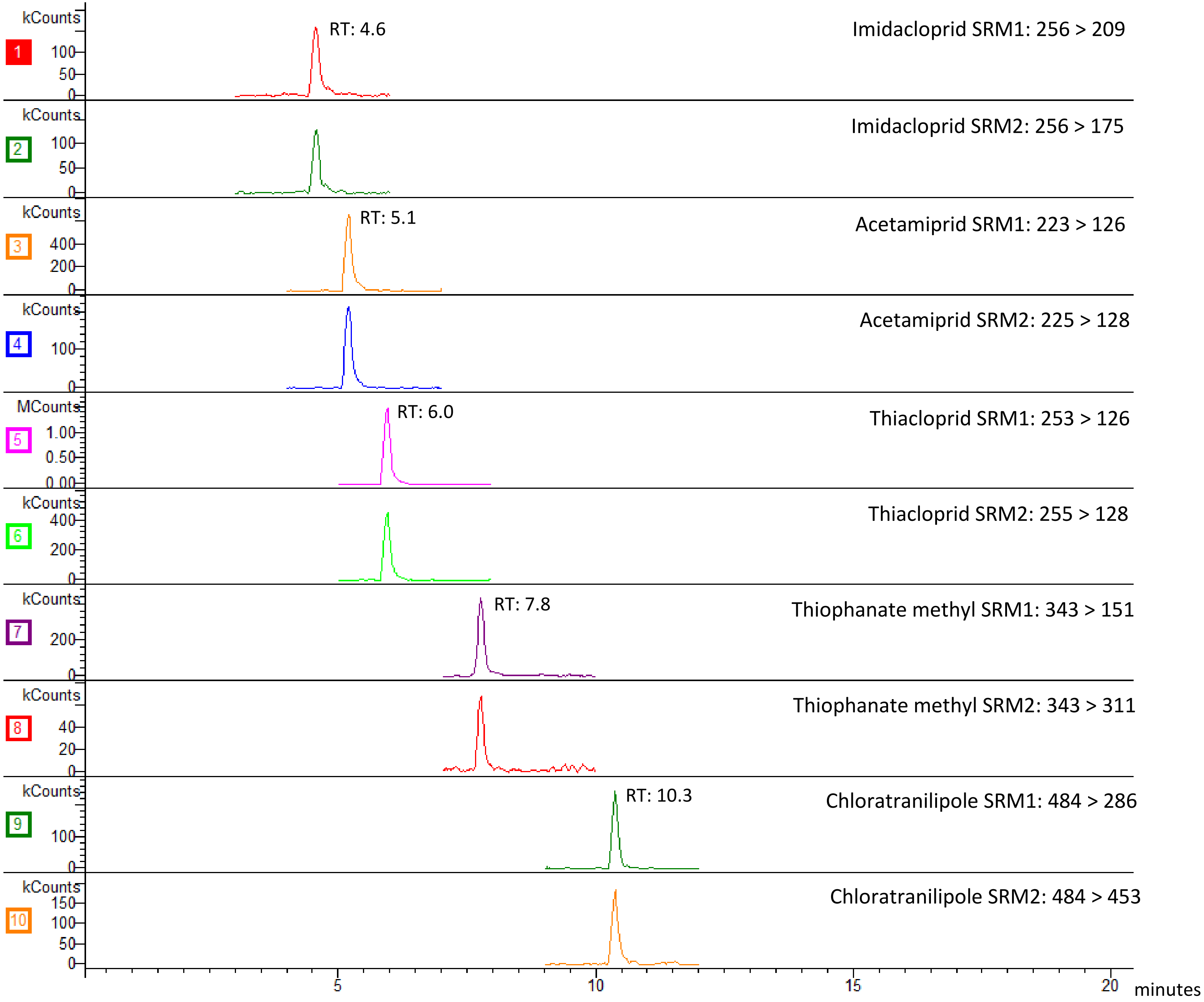

Figure S1 (iii). Quantification (SRM1) and Confirmation (SRM2) chromatograms of analytes Boscalid – Methoxyfenozide determined in Fraction C of sludge sample fortified at 10 ng/g.

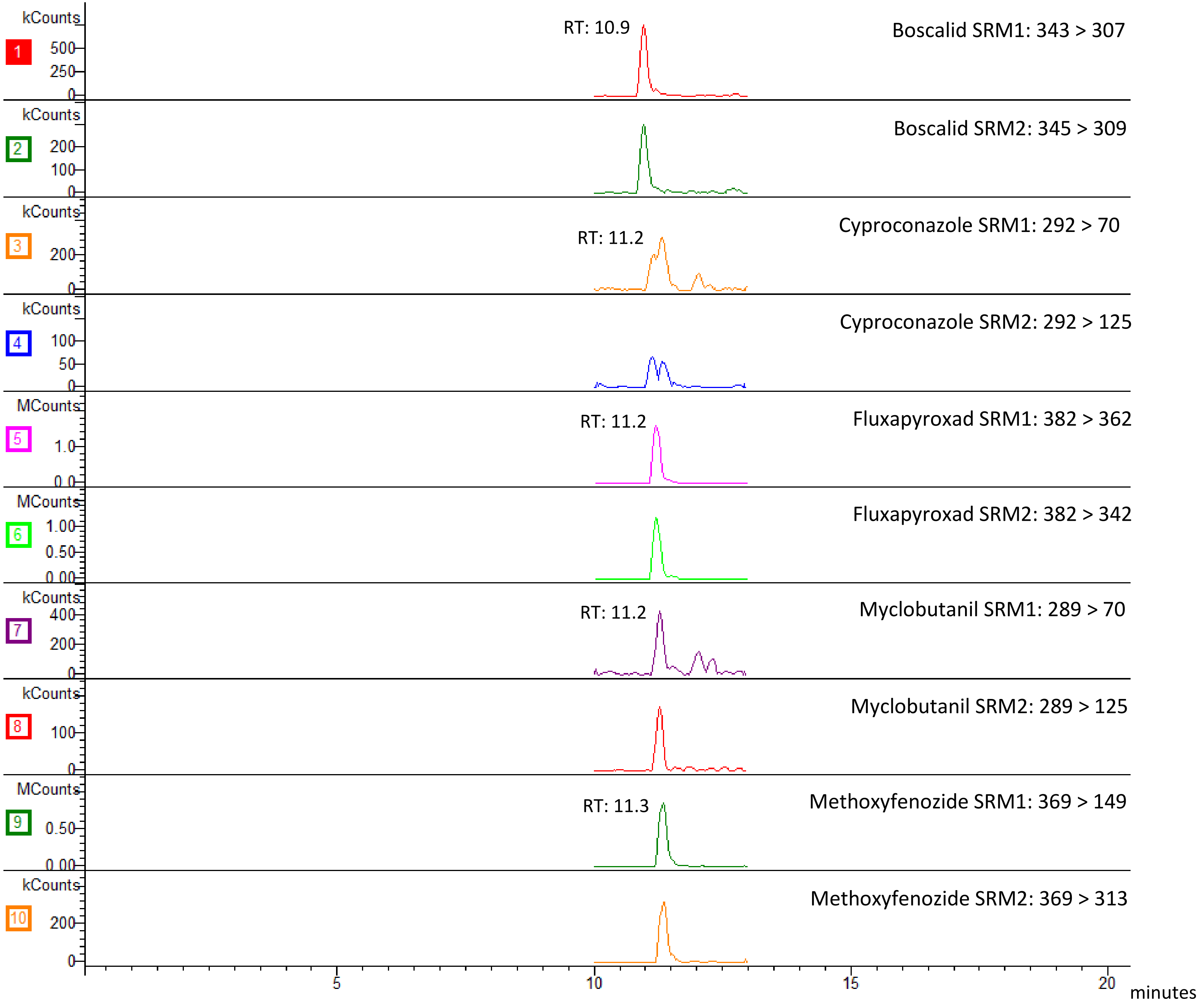

Figure S1 (iv). Quantification (SRM1) and Confirmation (SRM2) chromatograms of analytes Bupirimate – Fluopyram determined in Fraction C of sludge sample fortified at 10 ng/g.

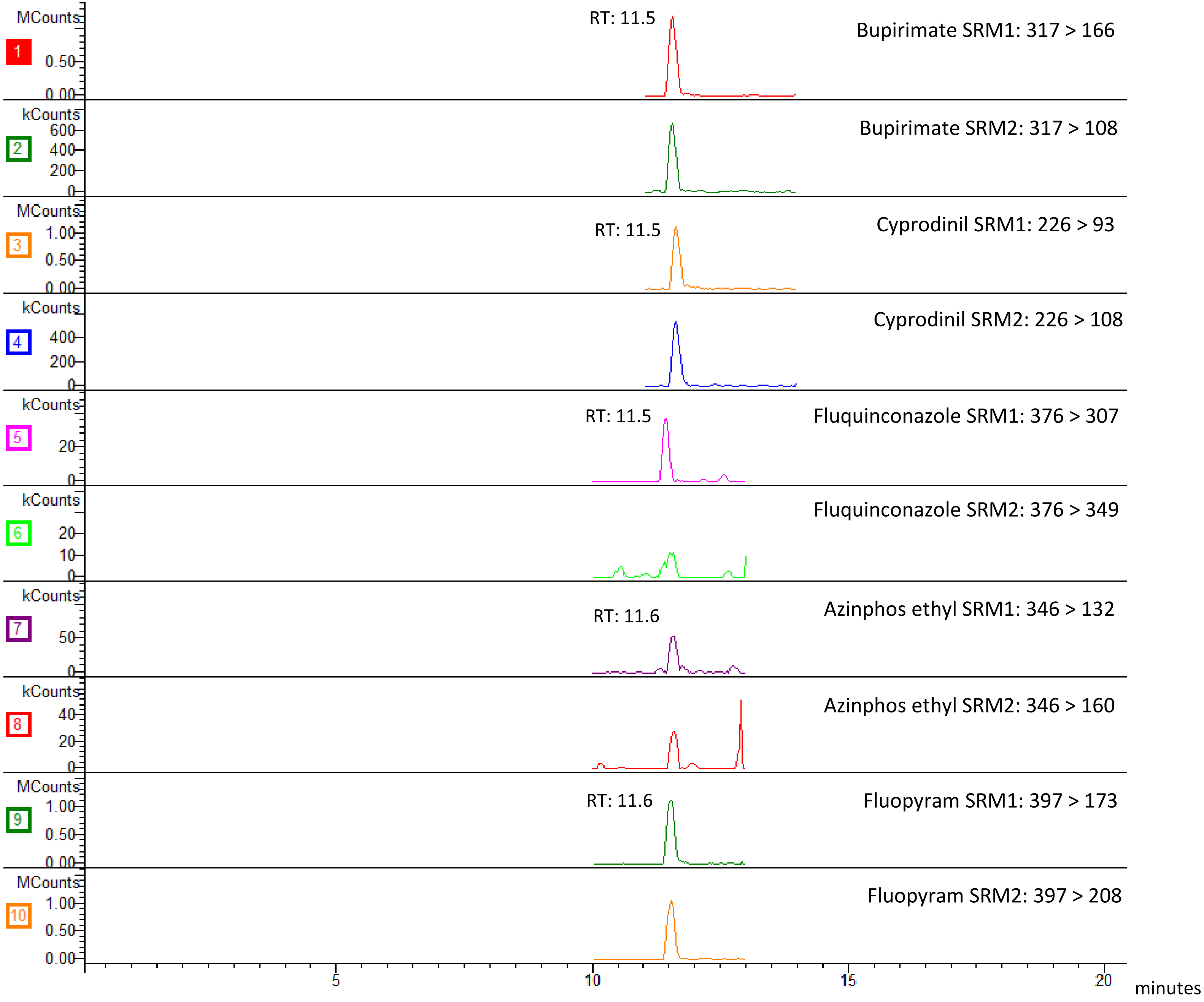

Figure S1 (v). Quantification (SRM1) and Confirmation (SRM2) chromatograms of analytes Fenbuconazole – Tebuconazole determined in Fraction C of sludge sample fortified at 10 ng/g.

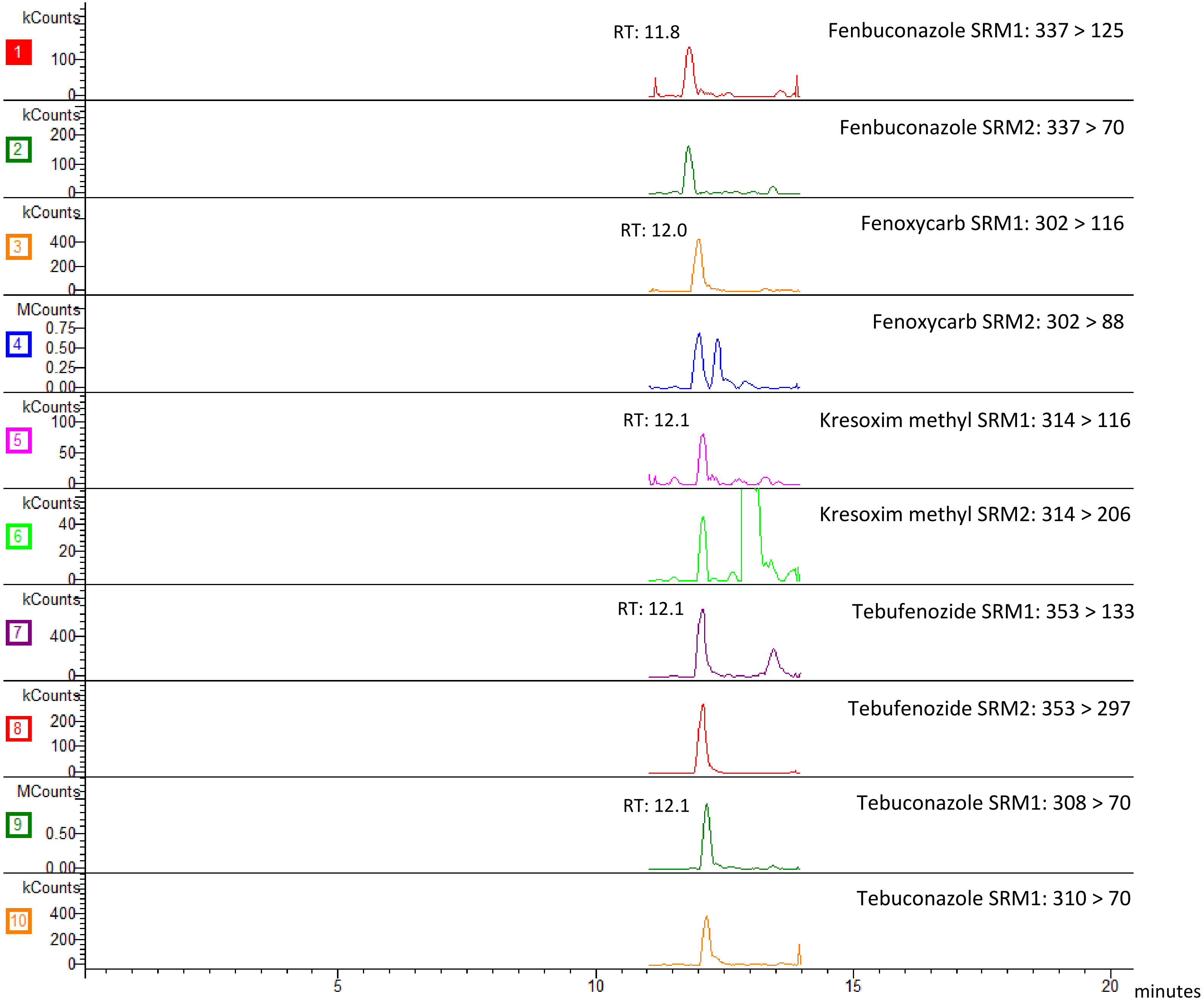

Figure S1 (vi). Quantification (SRM1) and Confirmation (SRM2) chromatograms of analytes Triphenyl-phosphate (I.S) – Difenoconazole determined in Fraction C of sludge sample fortified at 10 ng/g.

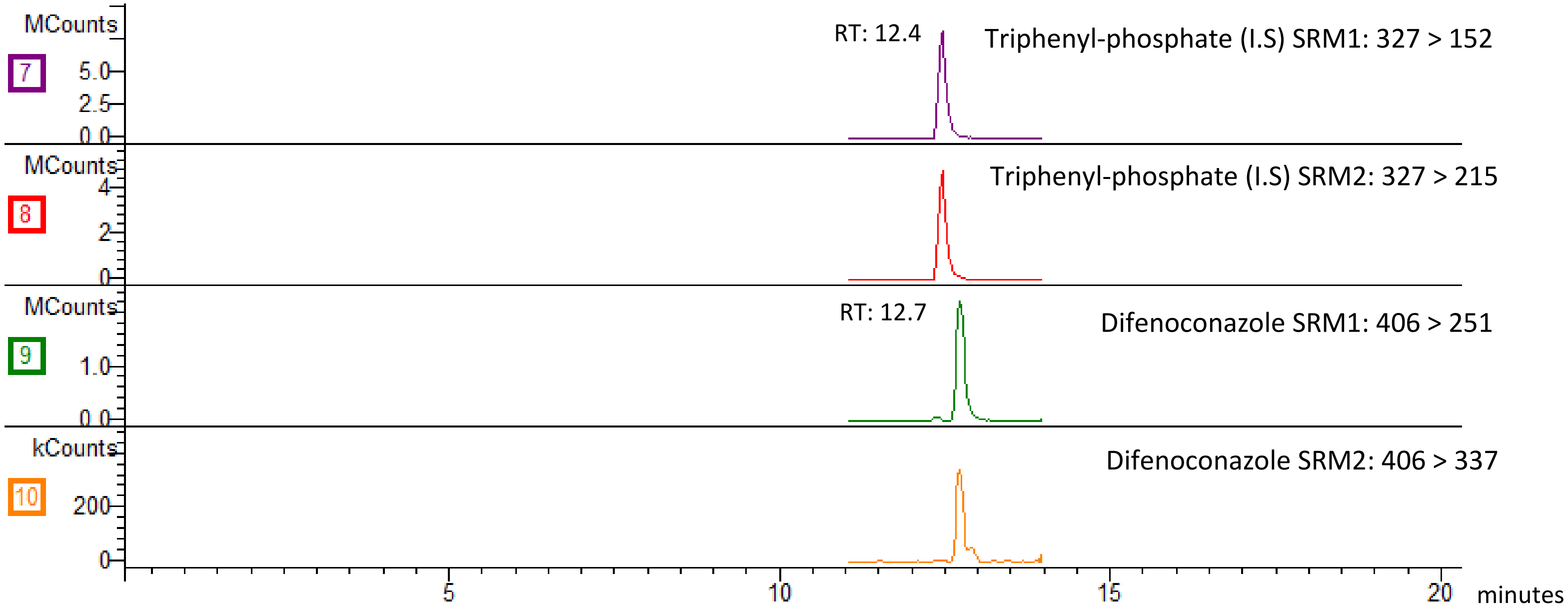

Figure S1 (vii). Quantification (SRM1) and Confirmation (SRM2) chromatograms of analyte Fludioxonil determined in Fraction C of sludge sample fortified at 10 ng/g, in negative polarity.

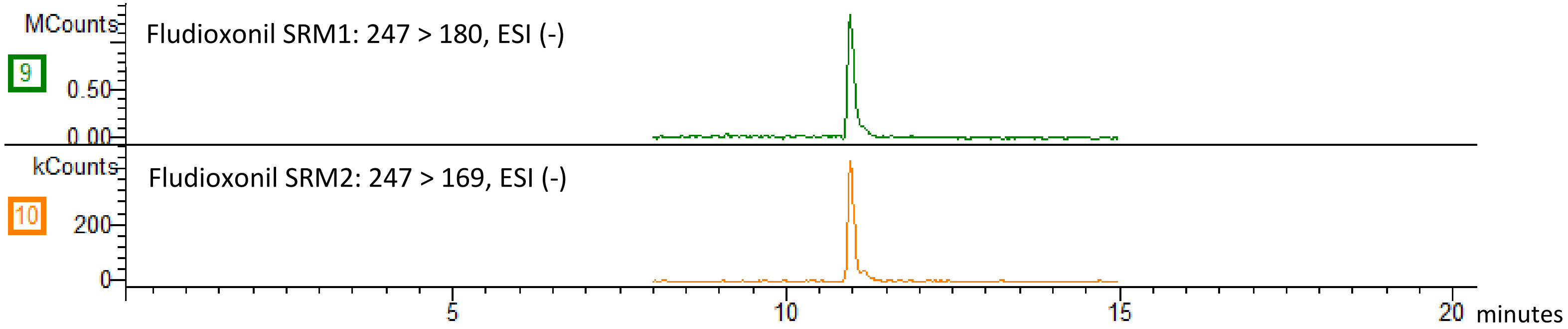

Figure S2 (i). Quantification (SRM1) and Confirmation (SRM2) chromatograms of analytes Imazalil – Triphenyl-phosphate (I.S) determined in Fraction B of sludge sample fortified at 10 ng/g.

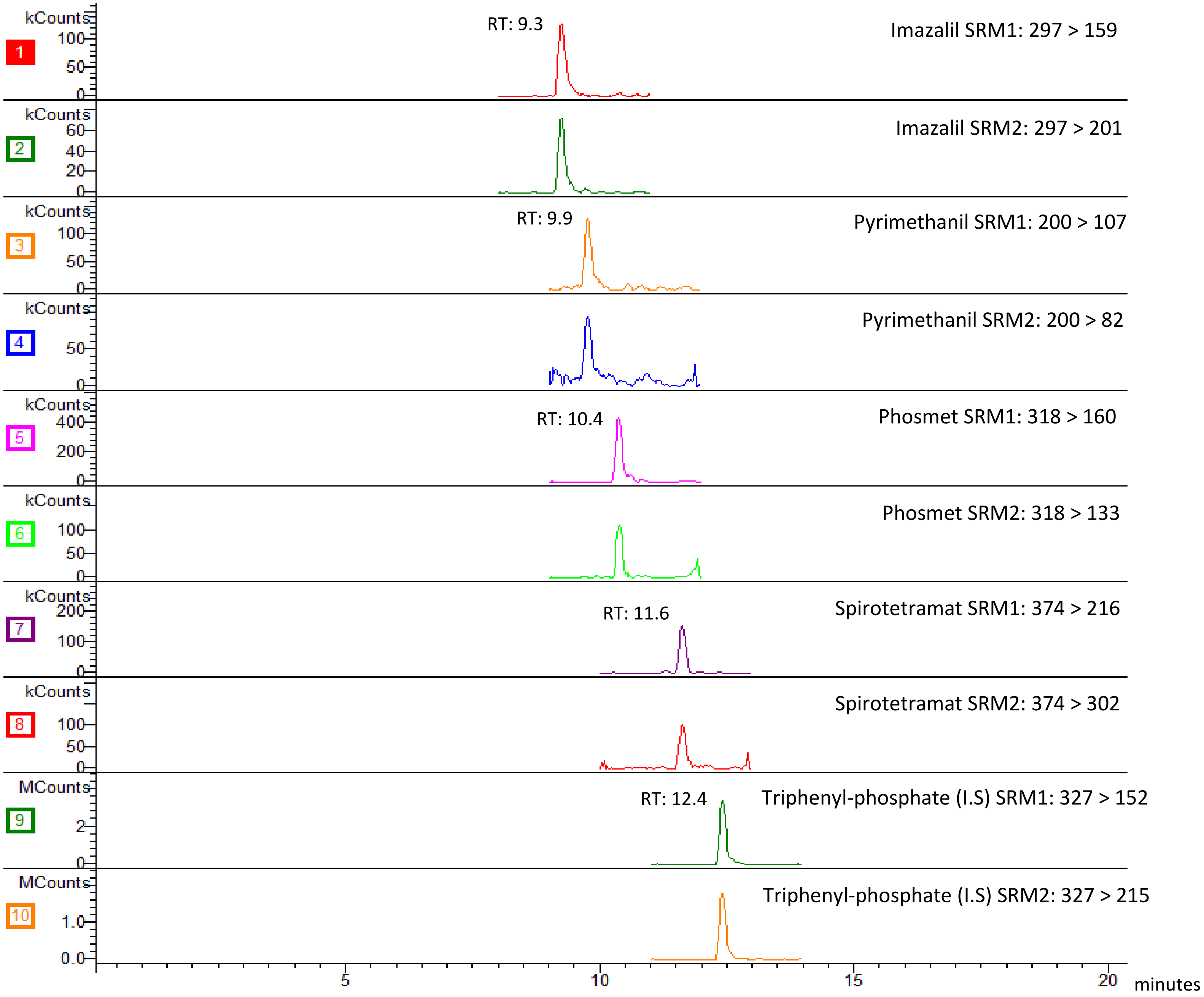

Figure S2 (ii). Quantification (SRM1) and Confirmation (SRM2) chromatograms of analytes Phosalone – Indoxacarb determined in Fraction B of sludge sample fortified at 10 ng/g.

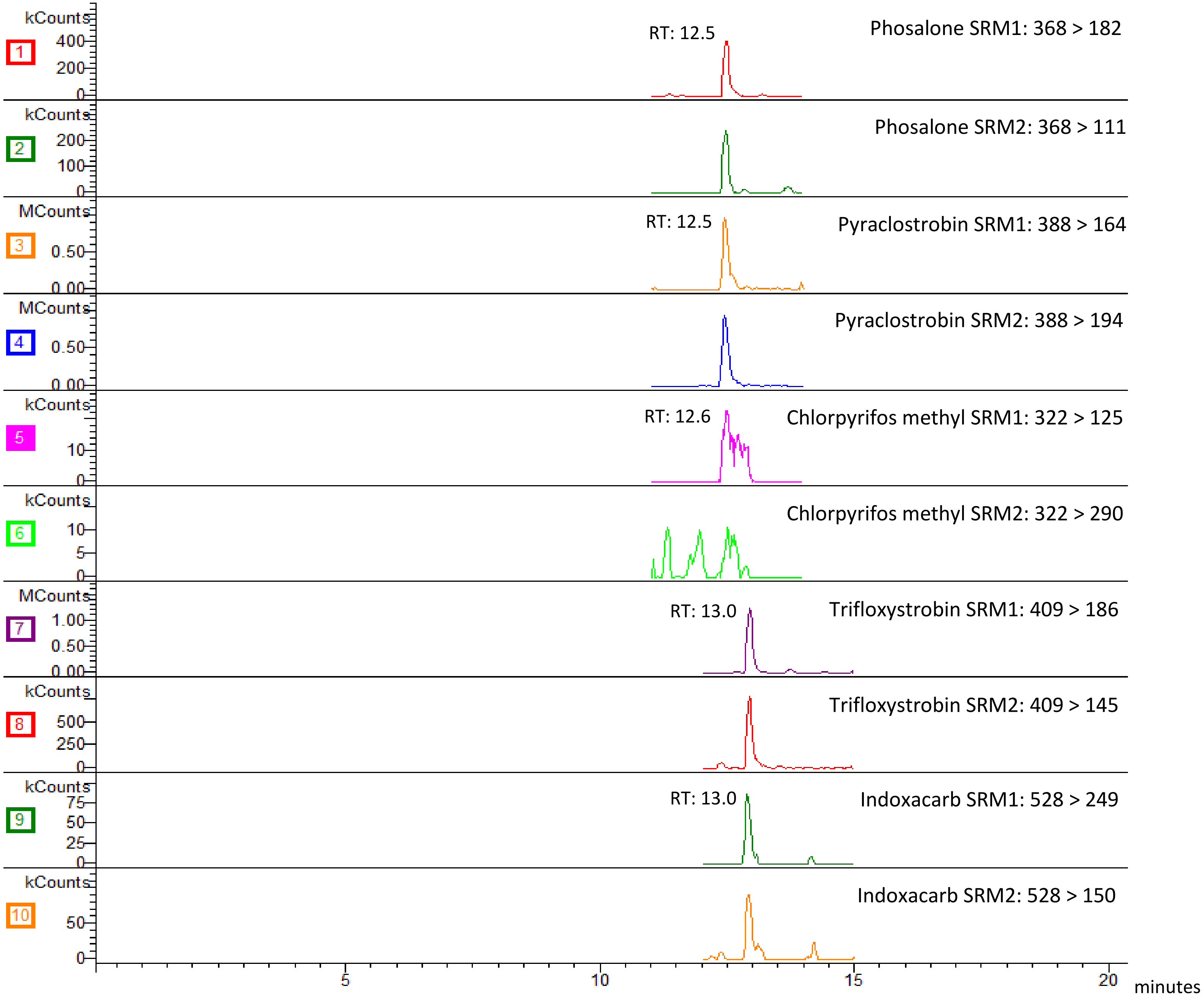

Figure S2 (iii). Quantification (SRM1) and Confirmation (SRM2) chromatograms of analytes Pyriproxyfen – b-Cyfluthrin determined in Fraction B of sludge sample fortified at 10 – 200 ng/g.

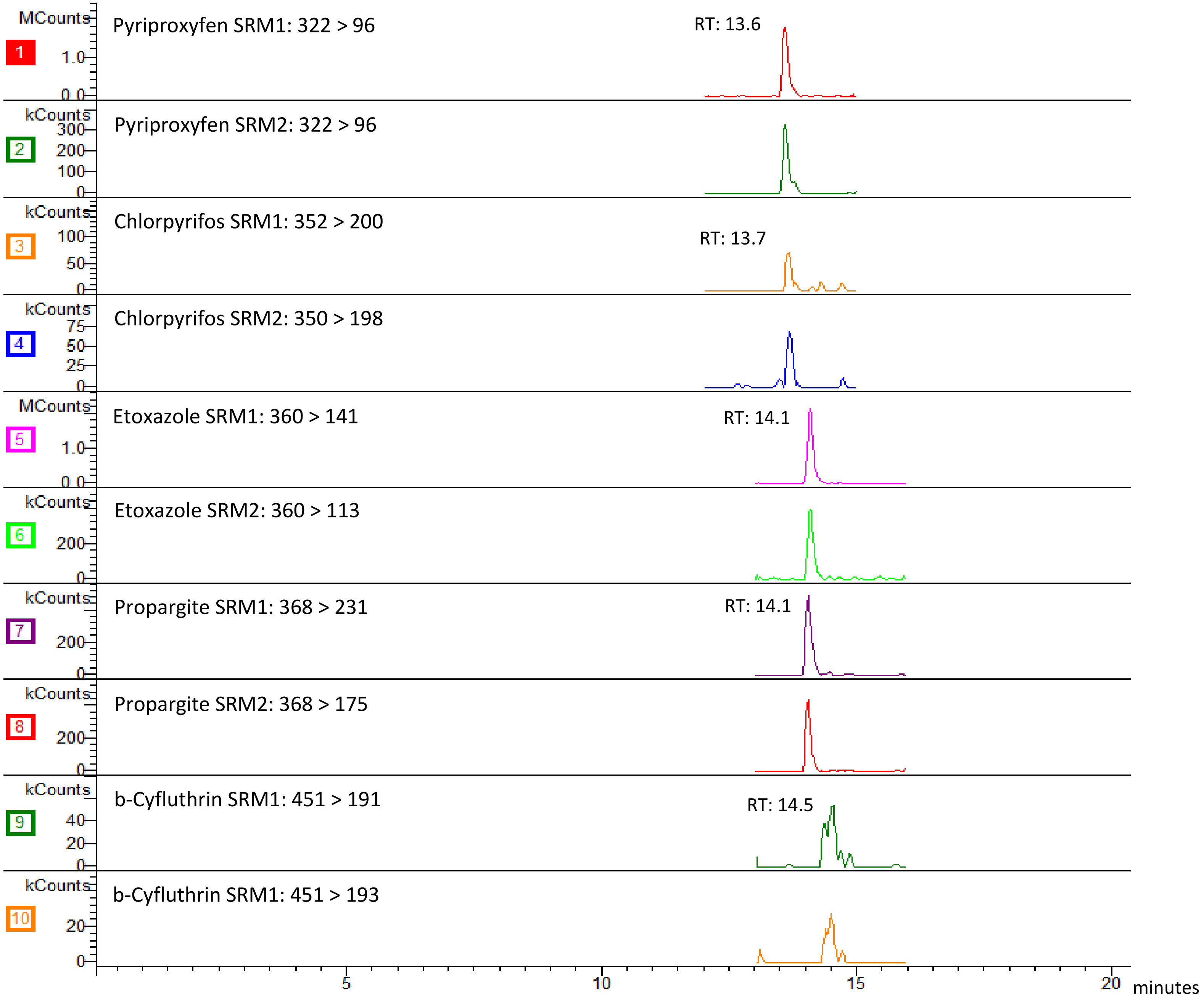

Figure S2 (iv). Quantification (SRM1) and Confirmation (SRM2) chromatograms of analytes Fenpyroximate – Etofenprox determined in Fraction B of sludge sample fortified at 10 – 50 ng/g.

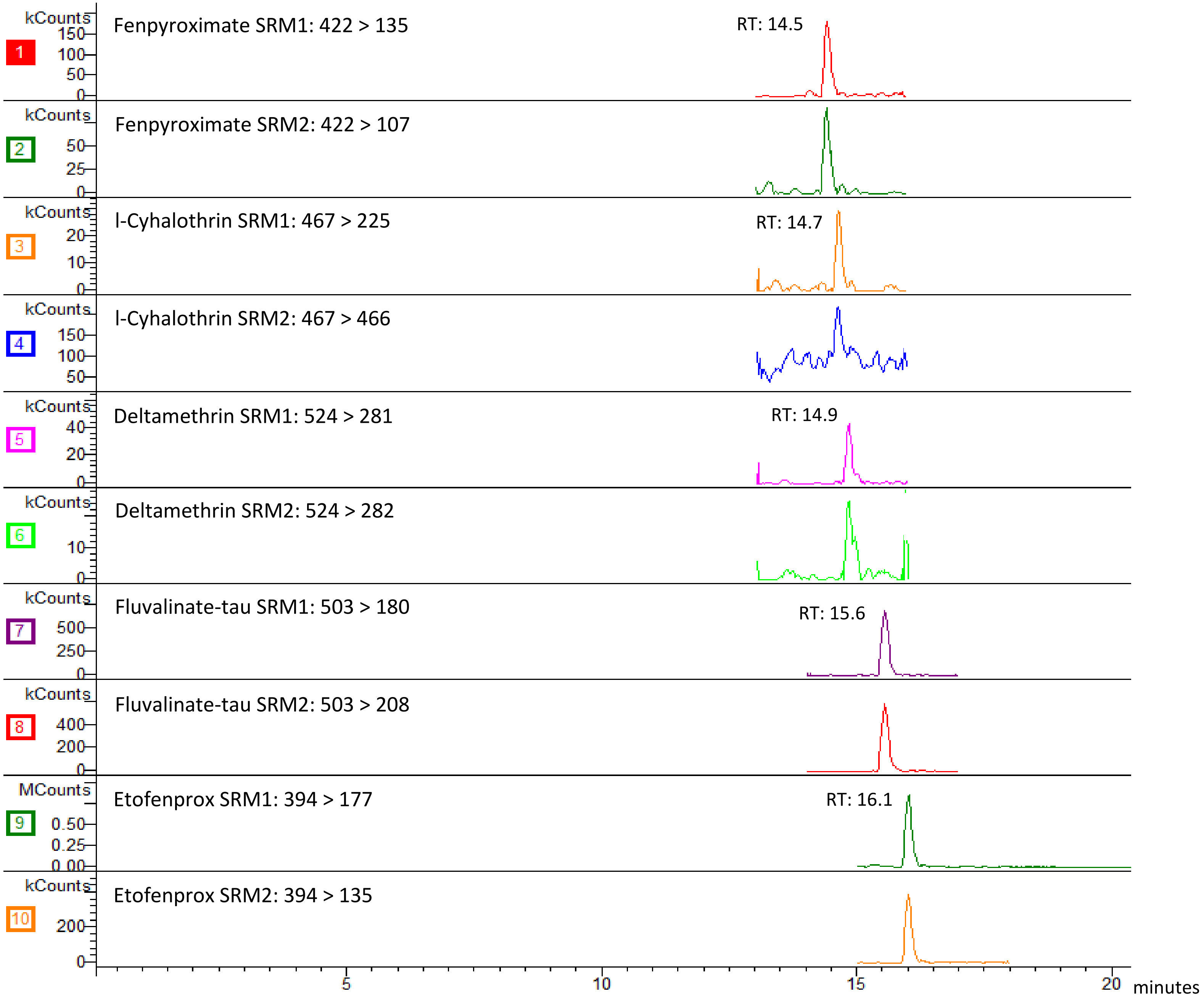

Figure S2 (v). Quantification (SRM1) and Confirmation (SRM2) chromatograms of analyte Bifenthrin determined in Fraction B of sludge sample fortified at 10 ng/g.

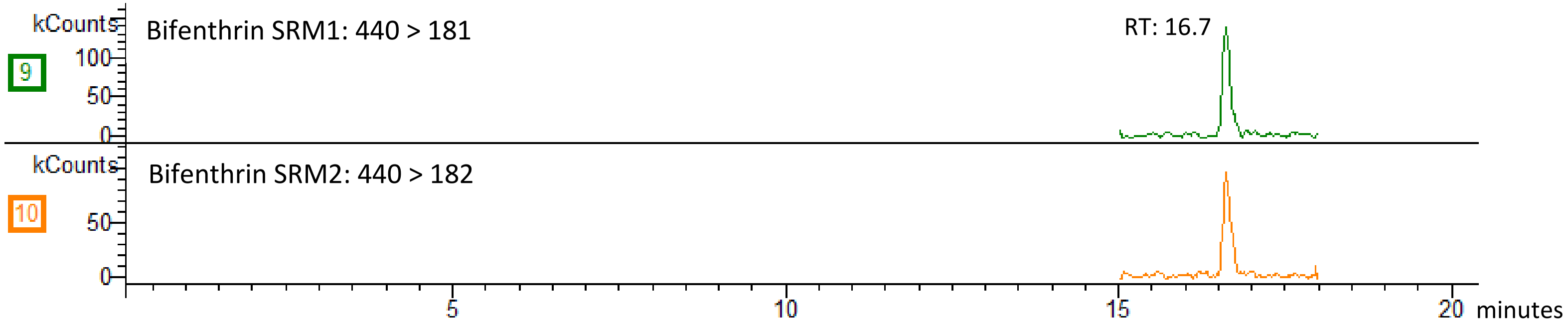

Supplement: Supplementary file 1 [file molecules-26-06888-s001.zip › molecules-1441978-supplementary.pdf]
